# Nortriterpenoids from the Fruiting Bodies of the Mushroom *Ganoderma resinaceum*

**DOI:** 10.3390/molecules22071073

**Published:** 2017-06-28

**Authors:** Xian-Qiang Chen, Ling-Xiao Chen, Jing Zhao, Yu-Ping Tang, Shao-Ping Li

**Affiliations:** 1 State Key Laboratory of Quality Research in Chinese Medicine, Institute of Chinese Medical Sciences, University of Macau, Macau 999078, China; yb27528@umac.mo (X.-Q.C.); yb47517@umac.mo (L.-X.C.); 2 Jiangsu Collaborative Innovation Center of Chinese Medicinal Resources Industrialization, Jiangsu Key Laboratory for High Technology Research of TCM Formulae, and National and Local Collaborative Engineering Center of Chinese Medicinal Resources Industrialization and Formulae Innovative Medicine, Nanjing University of Chinese Medicine, Nanjing 210023, China; yupingtang@njutcm.edu.cn

**Keywords:** *Ganoderma resinaceum*, nortriterpenoid, lucidones I–L, *α*-glucosidase

## Abstract

*Ganoderma resinaceum* is usually used as ethnomedicine for immune-regulation, hyperglycemia, and liver disease. To date, only a few chemical constituents have been reported from *G*. *resinaceum*. In this study, fifteen nortriterpenoids including six new nortriterpenoids (**1**–**6**) and nine known analogs (**7**–**15**), were separated and purified from the fruiting bodies of *G*. *resinaceum*. New compounds were identified as lucidone I (**1**), lucidone J (**2**), lucidone K (**3**), lucidone I (**4**), ganosineniol B (**5**), and ganosineniol C (**6**), based on analysis of extensive spectroscopic data (high resolution mass spectrometry (HRMS), nuclear magnetic resonance (NMR), infrared (IR), and ultraviolet (UV)). The known compounds were assigned as lucidone A (**7**), lucidone B (**8**), lucidone H (**9**), lucidone E (**10**), lucidone F (**11**), lucidone D (**12**), lucidone C (**13**), ganoderense F (**14**), and ganosineniol A (**15**), by comparing their spectroscopic data with those reported in the literature. Compounds **3**, **4**, and **7**–**13** were examined for *α*-glucosidase inhibitory activity and display no significant activity, but the finding may support that the side chain of ganoderma triterpenoids played an important role in *α*-glucosidase inhibitory activity.

## 1. Introduction

Nortriterpenoids, derived from lanostane-type triterpenoids due to degradation of side chain, are a class of secondary metabolites in *Ganoderma* [1,2]. Although substantial triterpenoids have been reported from *Ganoderma*, nortriterpenoid is rare. The common nortriterpenoids possess 24 or 27 carbons skeleton in *Ganoderma*, for example, lucidones A–H, and lucidenic acids A–N. However, two novel nortriterpenoids, methyl ganosinensate A and ganosinensic acid A, contain an unusual four-membered ring skeleton produced by a bond across C-1 to C-11 [3]. Nortriterpenoids isolated from *Ganoderma* showed a wide range of biological activities, such as anti-tumor [4,5,6], anti-inflammatory [7], neurotrophic [8], hepatoprotective [9], and anti-HIV-1 protease activities [10]. Hence, nortriterpenoids deserve our close attention due to its structure diversity and good model for the pharmaceutical field.

**Figure 1:**
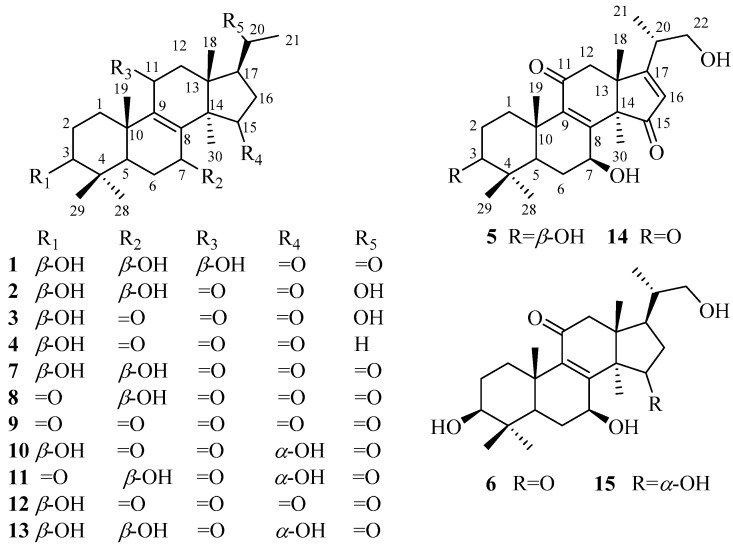
Structures of compounds **1**–**15**.

As a member of the genus *Ganoderma*, *G*. *resinaceum* has been used for immune-regulation, hyperglycemia, and liver disease [11]. The extract of *G*. *resinaceum* exhibited antimicrobial, antioxidant, and inhibitory activities against acetyl cholinesterase, tyrosinase, *α*-amylase and *α*-glucosidase [12]. At present, only 17 triterpenoids had been obtained from the fruiting bodies of *G*. *resinaceum*, whose biological properties included cytotoxicity [13] and hepatoprotective activities [9]. Obviously, the chemical constituents isolated from *G*. *resinaceum* and their biological activities have not been thoroughly characterized yet. Aiming to elucidate bioactive constituents from *G*. *resinaceum*, our team carried out phytochemical investigation on *G*. *resinaceum*. In this study, six new compounds (**1**–**6**) and nine known compounds (**7**–**15**) (Figure 1) were obtained from the fruiting bodies of *G*. *resinaceum*. The structures of the new compounds were elucidated by extensive spectroscopic data (HRMS, NMR, IR, and UV). The known compounds were identified by comparison of MS and 1 D NMR spectroscopic data with those reported in the literature. Compounds **3**, **4**, and **7**–**15** were evaluated for inhibitory activity against *α*-glucosidase and display no significant activity, however this finding may support that the side chain of ganoderma triterpenoids is critical for *α*-glucosidase inhibitory activity.

## 2. Results and Discussion

The 95% ethanol extract of *G. resinaceum* was partitioned with petroleum ether, EtOAc, and *n*-BuOH, successively. The triterpene-containing EtOAc and *n*-BuOH fractions were repeatedly purified by column chromatography over silica gel, MCI gel, ODS gel, Sephadex LH-20, and preparative HPLC to afford 20 nortriterpenoids, including six new compounds (**1**–**6**) and nine known compounds (**7**–**15**). By comparison of spectroscopic data with those reported in literature, the known compounds were identified as lucidone A (**7**) [14], lucidone B (**8**) [14,15], lucidone H (**9**) [16], lucidone E (**10**) [9], lucidone F (**11**) [9], lucidone D (**12**) [9], lucidone C (**13**) [15], ganoderense F (**14**) [17], and ganosineniol A (**15**) [18]. Structural elucidation of new compounds was as follows.

Compound **1** was obtained as white amorphous powder. Its molecular formula was established as C_24_H_36_O_5_ by electrospray ionization (ESI)-HRMS at *m*/*z* 403.2484 [M − H]^−^ (calcd. for C_24_H_35_O_5_, 403.2484). Its IR spectrum showed the presence of hydroxy group (3429 cm^−1^) and carbonyl group (1717 cm^−1^). ^1^H-NMR spectrum showed the presences of six singlet methyl signals [δ_H_ 0.77, 0.79, 0.90, 1.08, 1.21, and 2.13 (each 3H, s)]. Heteronuclear single quantum coherence (HSQC) and ^13^C-NMR spectra displayed 24 carbon resonances, including six methyls, five methylenes, five methines including three oxygenated carbon resonances at δ_C_ 79.5, 66.9 and 65.3, and eight quaternary carbons including a tetrasubstituted olefinic carbon δ_C_ 136.8 and 146.2, and two ketone carbons δ_C_ 220.2 and 209.3. The above spectroscopic data suggested **1** to be nortriterpenoid similar to lucidone A (**7**). However, a comparison of their ^13^C-NMR data showed that **1** possesses an additional oxygenated methine at δ_C_ 65.3 but absent of one ketone group signal at δ_C_ 196.7 in **7**. Moreover, the chemical sifts of tetrasubstituted olefinic carbons of **1** in ^13^C-NMR spectrum were significantly different from those of **7** due to the disappearance of α,β-unsaturated ketone conjugated system at C-8/C-9/C-11. Accordingly, it was assumed that **1** was the 11-OH derivative of **7**. The 11-OH was further confirmed on the basis of the heteronuclear multiple bond correlation (HMBC) correlations from H-11 (δ_H_ 4.48) to C-8 (δ_H_ 136.8), C-9 (δ_C_ 146.2) and C-13 (δ_C_ 43.8), and H-12β (δ_H_ 2.00) to C-11 (δ_H_ 65.3), and the hydrogen-hydrogen correlation spectroscopy (^1^H–^1^H COSY) correlation of H-11 (δ_H_ 4.48) with H-12α (δ_H_ 2.51). Finally, the planar structure of **1** was established by 1D and 2D NMR spectra.

**Figure 2:**
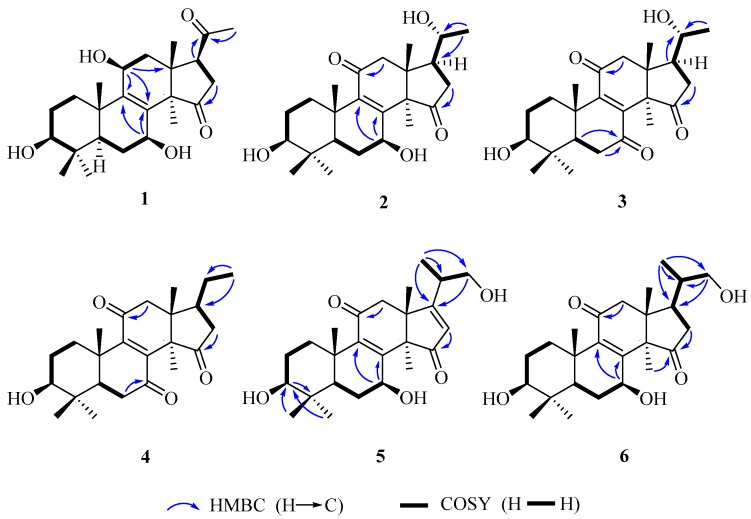
The key HMBC, and ^1^H–^1^H COSY correlations of **1**–**6**.

The relative configuration of **1** was established by analyses of rotating-frame nuclear overhauser effect correlation spectroscopy (ROESY) experiment and coupling constant. The ROESY correlations (Figure 2) of H-30 (δ_H_ 1.08) with H-12*α* (δ_H_ 2.51) and H-7 (δ_H_ 4.54), H-12α with H-11 (δ_H_ 4.48), and H-3 (δ_H_ 3.08) with H-28 (δ_H_ 0.90) and H-5 (δ_H_ 0.93) suggested 3-, 7- and 11-OH to be β-orientation, which were also supported by the larger coupling constant (Table 1). Thus, compound **1** was elucidated as 3β,7β,11β-trihydroxy-4,4,14α-trimethyl-15,20-dioxo-5α-pregn-8-ene, named lucidone I.

**Figure 3:**
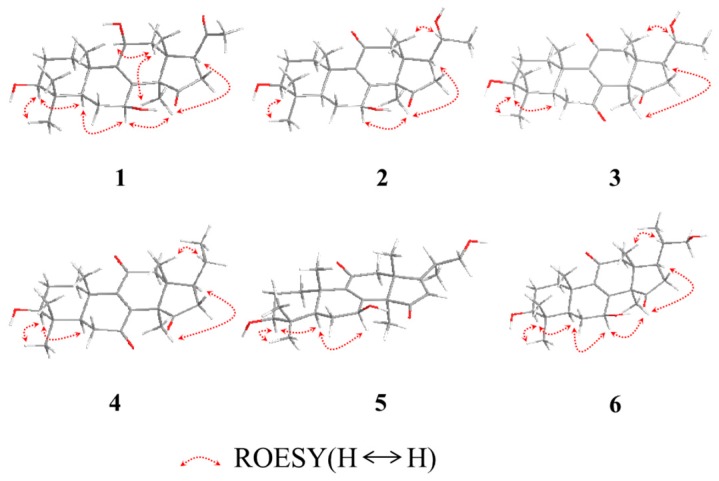
The key ROESY correlations of **1**−**6**.

Compound **2** was isolated as white powder. Its molecular formula was determined to be C_24_H_35_O_5_ due to HRESIMS at *m*/*z* 403.2481 [M − H]^−^ (calcd. for C_24_H_35_O_5_, 403.2484). Detail analyses of ^13^C-NMR (Table 1) and HSQC spectra of **2** showed that the structure of **2** was highly similar as that of **7**, except for the presence of an additional oxymethine and the absence of one ketone group in **2**. Moreover, ^1^H-NMR spectrum exhibited that **2** had one doublet methyl signal, whereas **7** did not, suggesting the hydroxy group located at C-20 in **2**, which was supported by the HMBC correlations from H-21 (δ_H_ 1.20) to C-20 (δ_C_ 69.9) and C-17 (δ_C_ 48.9), as well as the ^1^H–^1^H COSY correlations of H-20 (δ_H_ 3.79) with H-21 (δ_H_ 1.20) and H-17 (δ_H_ 2.28). The 3- and 7-hydroxy groups were assigned as β- and β-orientation, respectively, on the basis of ROESY correlations of H-3 with H-28 and H-5, H-7 with H-5 and H-30. The ROESY correlations of H-17 with H-30, and H-20 with H-18 (Figure 3) assigned the relative configuration of hydroxy attached to C-20 as α-orientation. Consequently, compound **2** was established as 3β,7β,20α-trihydroxy-4,4,14α-trimethyl-11,15-dioxo-5α-pregn-8-ene, named lucidone J.

Compound **3** was obtained as pale yellow powder. It had the molecular formula of C_24_H_34_O_5_ deduced from HRESIMS at *m*/*z* 401.2352 [M − H]^−^ (calcd. for C_24_H_33_O_5_, 401.2333). The IR and UV spectra revealed the presence of hydroxy group (3484 cm^−1^), carbonyl group (1722 cm^−1^) and α,β-unsaturated carbonyl group (1689 cm^−1^ and 259 nm). Comparison of spectroscopic data of **3** with that of **2** displayed their structural similarities except for the presence of ketone group to replace oxygenated methine. The ketone group at C-7 was confirmed by the key HMBC correlations from H-5 (δ_H_ 1.50) and H-6 (δ_H_ 2.60 and 2.40) to C-7 (δ_C_ 202.3). The 20α-OH was confirmed on the basis of ROESY correlations of H-17 (δ_H_ 2.28) with H-30 (δ_H_ 1.52), and H-18 (δ_H_ 0.80) with H-20 (δ_H_ 3.65) (Figure 3). Accordingly, compound **3** was established as 3β,20α-dihydroxy-4,4,14α-trimethyl-7,11,15-trioxo-5α-pregn-8-ene, named lucidone K.

Compound **4** was obtained as pale yellow powder. Its molecular formula was assigned as C_24_H_34_O_4_ by HRESIMS at *m*/*z* 445.2583 [M + CH_3_COOH − H]^−^ (calcd. for C_26_H_37_O_6_, 445.2590). The hydroxy group (3441 cm^−1^), carbonyl group (1743 cm^−1^) and α,β-unsaturated carbonyl group (1680 cm^−1^) were observed in IR spectrum. The UV absorption at λ_max_ 260 nm showed the presence of α,β-unsaturated carbonyl. A comparison of NMR data of **4** with those of **3** revealed their structural similarities. The major differences were the existence of triplet methyl signal but absent of an oxygenated methine at δ_C_ 69.9, suggesting the disappearance of 20-OH in **4**. The HMBC correlations from H-21 (δ_H_ 0.99) to C-20 (δ_C_ 32.9) and C-17 (δ_C_ 46.2), combined with the ^1^H–^1^H COSY correlations of H-20 (δ_H_ 2.13) with H-17 (δ_H_ 2.34) and H-21 (δ_H_ 0.99), confirmed that the ethyl group was attached to C-17. The 3-OH was assigned as β-orientation by the ROESY correlations of H-3 with H-28 and H-5. Therefore, compound **4** was determined as 3β-hydroxy-4,4,14α-trimethyl-7,11,15-trioxo-5α-pregn-8-ene, named lucidone L.

Compound **5** was isolated as white powder. Its molecular formula was established as C_25_H_36_O_5_ based on HRESIMS at *m*/*z* 415.2488 [M − H]^−^ (calcd. for C_25_H_35_O_5_, 415.2484). The IR spectrum showed the presence of hydroxy group (3436 cm^−1^) and α,β-unsaturated carbonyl group (1664 cm^−1^). The UV absorption brand λ_max_ 244 nm also supported the presence of α,β-unsaturated carbonyl group. Detail comparison of 1 D NMR spectroscopic data with those of ganoderense F (**14**) suggested their structural similarities except that the hydroxy group at C-3 replaced the ketone group in **5**. The 3-OH was confirmed by the HMBC correlations from H-28 (δ_H_ 1.07) and H-29 (δ_H_ 0.88) to C-3 (δ_C_ 78.2), and H-3 (δ_H_ 3.26) to C-28 (δ_C_ 28.2) and C-29 (δ_C_ 15.6), as well as the ^1^H–^1^H COSY correlation of H-3 (δ_H_ 3.26) with H-2 (δ_H_ 1.70). The hydroxy groups at C-3 and C-7 were all assigned as β-orientation on the basis of the ROESY correlations (Figure 3) of H-3 (δ_H_ 3.26) with H-28 (δ_H_ 1.07) and H-5 (δ_H_ 0.98), and H-7 (δ_H_ 4.84) with H-5 (δ_H_ 0.98) and H-30 (δ_H_ 1.58), Thus, compound **5** was elucidated as 20-carbinol-3β,7β-dihydroxy-4,4,14α-trimethyl-11,15-trioxo-5α-pregn-8,16-dien, named ganosineniol B.

Compound **6** was obtained as white powder. Its molecular formula was determined as C_25_H_38_O_5_ by HRESIMS at *m*/*z* 417.2629 [M − H]^−^ (calcd. for C_25_H_37_O_5_, 417.2641). The presence of hydroxy group (3432 cm^−1^), carbonyl group (1722 cm^−1^) and α,β-unsaturation group (1655 cm^−1^ and 254 nm) were confirmed by IR and UV spectra. Detail analyses of ^1^H, ^13^C-NMR and HSQC spectra revealed that the structure of **6** was highly similar to that of ganosineniol A (**15**). The difference between those two constituents was that one oxymethine was converted into ketone in **6**. The ^13^C-NMR spectrum of **6** did not show any signal at around δ_C_ 72.6, but revealed an additional downfield shift at δ_C_ 218.9. Hence, it was inferred that the ketone was located at C-15. The hypothesis was proved by the key HMBC correlations from H-30 (δ_H_ 1.30) and H-16 (δ_H_ 2.68 and 2.10) to C-15 (δ_C_ 218.9). The ROESY correlations of H-3 (δ_H_ 3.08) with H-28 (δ_H_ 0.95) and H-5 (δ_H_ 0.87), and H-7 (δ_H_ 4.78) with H-5 (δ_H_ 0.87) and H-30 (δ_H_ 1.30) assigned 3-OH and 7-OH as β-orientation. Accordingly, compound **6** was determined as 20-carbinol-3β,7β-dihydroxy-4,4,14α-trimethyl-11,15-dioxo-5α-pregn-8-dien, named ganosineniol C.

**Figure 4:**
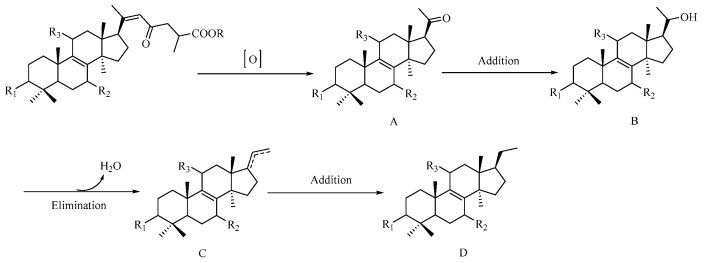
Plausible biogenetic pathway for C24 nortriterpenoids.

Triterpenoids, biogenetically derived via mevalonic acid pathway, are the main secondary metabolites of *Ganoderma*. The above-mentioned nortriterpenoids are considered to originate from triterpenoid due to the degradation of its side chain. A possible biogenetic pathway for C24 nortriterpenoids is proposed as shown in Figure 4. The precursor, Ganoderenic acid and its esterified derivatives, undergoes oxidation to yield intermediate A (**1**, and **7**–**13**). Compounds **7** and **12** were further transformed into **2** and **3**, respectively, through an addition reaction. Finally, compound **3** (intermediate B) generated **4** (D) via elimination reaction and addition reaction.

*G*. *resinaceum* has been used as ethnomedicine for lowering blood sugar in Nigeria [19]. However, it is unknown whether chemical constituents from *G*. *resinaceum* contribute to the traditional medicinal efficacy. Therefore, we considered carrying out α-glucosidase inhibitory assay of isolates from *G*. *resinaceum*. Triterpenoids from *Ganoderma* exhibited significant α-glucosidase inhibitory activity [20,21,22]. Nortriterpenoids from *G. resinaceum* are considered to originate from triterpenoids due to the degradation of its side chain on basis of analysis of possible biogenetic pathway. Aiming to explore biological active constituents and structure–activity relationship of terpenoids from *Ganoderma*, compounds **3**, **4**, and **7**–**13** were evaluated for α-glucosidase inhibitory activity. Their inhibitions were less than 50% at the concentration of 3 mM (see Supplementary Materials, Table S1). All isolates measured showed lower inhibitory activity compared with positive drug acarbose (IC_50_ value 2.76 mM), hence, their IC_50_ value were not measured. Our bioactivity studies may support previous research results that the side chain played an important role in α-glucosidase inhibitory activity of ganoderma triterpenoids, especially the presence of carboxyl acid group [22]. For example, the only difference between ganoderic acid A and **11** was that ganoderic acid A had aliphatic side chain, whereas **11** did not. Ganoderic acid A displayed the same inhibitory activity as positive drug acarbose [22], however, Compound **11** was almost inactive (16% inhibition at the concentration of 3 mM).

## 3. Materials and Methods

### 3.1. General Experimental Procedures

NMR spectra data were recorded on a Bruker ascend 600 spectrometer (Bruker, Karlsruhe, Germany) with TMS used as a reference. Optical rotations were measured on PerkinElmer Model 341 polarimeter (PerkinElmer, Waltham, MA, USA). UV spectrum data were acquired using HACH DR6000 UV-visible spectrophotometer (Hach, Loveland, CO, USA). IR spectra were recorded as KBr disks on PerkinElmer Spectrum 100 Series FT-IR spectrometers (PerkinElmer, Waltham, MA, USA). HRESIMS data were obtained on a LTQ Orbitrap XL™ Hybrid Ion Trap-Orbitrap FT-MS spectrometer (Thermo, Waltham, MA, USA). TLC was carried out on silica gel GF_254_ plates (Yantai Institute of Chemical industry, Yantai, China) and spots were visualized by UV light (254 and/or 365 nm) and spraying with 10% H_2_SO_4_ followed by heating. Column chromatography was carried out using silica gel (Qingdao Haiyang Chemical Co., Ltd., Qingdao, China), MCI gel (CHP-20P, 75–150 μm, Mitsubishi Chemical Corporation, Tokyo, Japan), ODS (35–70 μm, Grace, Maryland, MD, USA), and Sephadex LH-20 (GE Healthcare Bio-Science AB, Uppsala, Sweden) as packing materials. Semi-preparative HPLC was performed on a Shimadzu instrument (Shimadzu, Tokyo, Japan) coupled to CBM-20A system controller, LC-20AP pump, SPD-M20A Photodiode Array Detector and SIL-10AP autosampler and equipped with a Shimadzu PRC-ODS column (250 mm × 20 mm i.d., 15 μm).

### 3.2. Fungal Material

Fruiting bodies of *G*. *resinaceum* were purchased in December 2014 from the Haikou Ruizhitang Wild Lingzhi Co., Ltd. in Hainan province, China, and identified as *G*. *resinaceum* Boud by one of author (Prof. S. P. Li). A voucher specimen (No. ICMS-SQC-20141201) has been deposited at Institute of Chinese Medical Sciences, University of Macau.

### 3.3. Extraction and Isolation

The dried fruiting bodies of *G. resinaceum* (48 kg) were powdered and extracted with 95% EtOH (600 L) twice under reflux for 2 h. The combined extracts were concentrated under vacuum to afford the crude extract (2.6 kg). After remove of solvent, the extract was dispersed in water and partitioned with petroleum ether, EtOAc and *n*-BuOH, successively. The HPLC and TLC profiling of EtOAc and *n*-BuOH extract showed that their constituents were similarities, hence, the EtOAc extract was merged with the *n*-BuOH extract. The mixture of EtOAc and *n*-BuOH extract was subjected to silica gel column chromatography (CC) eluted with a gradient of CHCl_3_–MeOH (100:0–0:100, *v*/*v*) to obtain three fractions (E1–E3). E1 (358 g) was separated on silica gel CC eluted with petroleum ether–acetone (100:0–0:100, *v*/*v*) to obtain four subfractions (E11–E14).

E12 (135.0 g) was chromatographed on silica gel CC eluted with petroleum ether–EtOAc (10:1–0:1, *v*/*v*) to yield fractions E12A and E12B. E12B (55 g) was subjected to MCI gel CC eluted with MeOH–H_2_O (70:30–100:0, *v*/*v*) to yield seven fractions (E12B1–E12B7). E12B3 was separated over silica gel CC eluted with CHCl_3_–MeOH (30:1, *v*/*v*) and then purified over semi-preparative HPLC using MeCN–H_2_O (30:70, *v*/*v*) as mobile phase to afford **7** (151.0 mg) and **10** (118.0 mg). E12B4 was subjected to silica gel CC eluted with CHCl_3_–MeOH (30:1, *v*/*v*) to obtain two fractions (E12B41 and E12B42). The fraction E12B42 was chromatographed over Sephadex LH-20 gel CC eluted with MeOH to yield two fractions (E12B42A and E12B42B). E12B42B was fractionated on semi-preparative HPLC eluted with MeCN–H_2_O (31:69, *v*/*v*) to afford **8** (40.0 mg), **9** (23.0 mg), **11** (110.0 mg), and **14** (35.0 mg).

E13 (103.5 g) was chromatographed over silica gel CC eluted with CHCl_3_–acetone (10:1–0:1) to yield three fractions (E13A–E13C). E13A was fractionated on MCI gel CC eluted with MeOH–H_2_O (50:50–100:0, *v*/*v*) to obtain three fractions (E13A1–E13A3). E13A1 was chromatograph over silica gel CC eluted with CHCl_3_–acetone (10:1–8:1) to yield two fractions (E13A11 and E13A12). Compound **12** (2.1 g) was obtained from E13A11 which was separated on Sephadex LH-20 gel CC. E13A12 was subjected to Sephadex LH-20 gel CC eluted with CHCl_3_–MeOH (1:1, *v*/*v*) and further purified over semi-preparative HPLC eluted with MeCN–H_2_O (35:65, *v*/*v*) to obtain **5** (6.0 mg). E13B was fractionated into four fractions (E13B1–E13B4) by ODS CC eluted with MeOH–H_2_O (50:50–80:20, *v*/*v*). E13B1 was purified over Sephadex LH-20 gel CC eluted with CHCl_3_–MeOH (1:1, *v*/*v*) and then semi-preparative HPLC with MeCN–H_2_O (32:68, *v*/*v*) to afford **13** (115.0 mg) and **3** (80.1 mg). E13C was chromatographed over MCI gel CC eluted with MeOH–H_2_O (40:60–80:20, *v*/*v*) to yield E13C1 and E13C2. E13C1 was fractionated on ODS CC eluted with a gradient of MeOH–H_2_O (50:50–80:20, *v*/*v*) to afford five fractions (E13C11–E13C15). Compound **1** (3.6 mg) was obtained from E13C13 which was subjected to Sephadex LH-20 gel CC and further semi-preparative HPLC eluted with MeCN–H_2_O (32:68, *v*/*v*). E13C14 was purified over ODS CC eluted with MeOH–H_2_O (50:50, *v*/*v*) and then semi-preparative HPLC with a MeCN–H_2_O (32:68, *v*/*v*) to afford **2** (5.1 mg) and **6** (35.0 mg). 

E2 (102.0 g) was chromatographed over silica gel CC eluted with CHCl_3_–acetone (5:1–0:1, *v*/*v*) to obtain E21 and E22. E21 (75.0 g) was separated on ODS CC eluted with MeOH–H_2_O (40:60–70:30, *v*/*v*) to afford four fractions (E21A–E21D). E21C was separated on ODS CC eluted with MeOH–H_2_O (40:60, *v*/*v*) to afford three fractions (E21C1–E21C3). E21C1 was subjected to Sephadex LH-20 gel CC using MeOH as mobile phase and further semi-preparative HPLC eluted with MeCN–H_2_O (31:69, *v*/*v*) to obtain **4** (40.0 mg). E22 (15.0 g) was fractionated on MCI gel CC eluted with MeOH–H_2_O (50:50–80:20, *v*/*v*) to yield E22A and E22B. E22A was separated on silica gel CC with an isocratic CHCl_3_–acetone (2:1, *v*/*v*) to obtain three fractions (E22A1–E22A3). Compound **15** (18.1 mg) was obtained from E22A2 by semi-preparative HPLC eluted with MeCN–H_2_O (30:70, *v*/*v*).

#### 3.3.1. *Lucidone I* (**1**)

White powder; ${\left[\alpha \right]}_{D}^{20}$ +67.4 (*c* 0.27, MeOH); UV (MeOH) λ_max_ (log ε) 204 (3.78), 253 (3.41) nm; IR (KBr) *v*_max_ 3429, 2691, 2929, 2871, 1717, 1639, 1453, 1383, 1261, 1181 cm^−1^; ^1^H- and ^13^C-NMR spectroscopic data see Table 1; HRESIMS *m*/*z* 403.2484 [M − H]^−^ (calcd. for C_24_H_35_O_5_, 403.2484).

#### 3.3.2. *Lucidone J* (**2**)

White powder; ${\left[\alpha \right]}_{D}^{20}$ +80.7 (*c* 0.38, MeOH); UV (MeOH) λ_max_ (log ε) 202 (3.45), 253 (3.76) nm; IR (KBr) *v*_max_ 3432, 2964, 2925, 2866, 1731, 1654, 1457, 1383, 1306, 1172, 1058, 1035 cm^−1^; ^1^H- and ^13^C-NMR spectroscopic data see Table 1; HRESIMS *m*/*z* 403.2481 [M − H]^−^ (calcd. for C_24_H_35_O_5_, 403.2484).

#### 3.3.3. *Lucidone K* (**3**)

Pale yellow powder; ${\left[\alpha \right]}_{D}^{20}$ +105.8 (*c* 0.17, MeOH); UV (MeOH) λ_max_ (log ε) 201 (3.53), 259 (3.68) nm; IR (KBr) *v*_max_ 3484, 2982, 2932, 2862, 1742, 1689, 1457, 1426, 1379, 1338, 1300, 1232, 1200, 1163, 1082, 1034, 976 cm^−1^; ^1^H- and ^13^C-NMR spectroscopic data see Table 1; HRESIMS *m*/*z* 401.2352 [M − H]^−^ (calcd. for C_24_H_33_O_5_, 401.2333). 

#### 3.3.4. *Lucidone L* (**4**)

Pale yellow powder; ${\left[\alpha \right]}_{D}^{20}$ +88.3 (*c* 0.74, MeOH); UV (MeOH) λ_max_ (log ε) 202 (3.49), 260 (3.58) nm; IR (KBr) *v*_max_ 3441, 2974, 2935, 2875, 1743, 1680, 1461, 1383, 1199, 1088, 1035, 928 cm^−1^; ^1^H- and ^13^C-NMR spectroscopic data see Table 1; HRESIMS *m*/*z* 445.2583 [M + CH_3_COOH − H]^−^ (calcd. for C_26_H_37_O_6_, 445.2590).

#### 3.3.5. *Ganosineniol B* (**5**)

White powder; ${\left[\alpha \right]}_{D}^{20}$ +104.6 (*c* 0.26, MeOH); UV (MeOH) λ_max_ (log ε) 201 (3.62), 244 (3.83), 341(2.05) nm; IR (KBr) *v*_max_ 3436, 2967, 2928, 2872, 1664, 1599, 1457, 1379, 1247, 1171, 1102, 1037 cm^−1^; ^1^H- and ^13^C-NMR spectroscopic data see Table 2; HRESIMS *m*/*z* 415.2488 [M − H]^−^ (calcd. for C_25_H_35_O_5_, 415.2484).

#### 3.3.6. *Ganosineniol C* (**6**)

White powder; ${\left[\alpha \right]}_{D}^{20}$ +19.9 (*c* 1.44, MeOH); UV (MeOH) λ_max_ (log ε) 202 (3.44), 254 (3.66), 341(2.04) nm; IR (KBr) *v*_max_ 3432, 2968, 2928, 2866, 1722, 1655, 1459, 1380, 1270, 1176, 1034 cm^−1^; ^1^H and ^13^C-NMR spectroscopic data see Table 2; HRESIMS *m*/*z* 417.2629 [M − H]^−^ (calcd. for C_25_H_37_O_5_, 417.2641).

### 3.4. Activity Assay

α-Glucosidase inhibitory activity was examined by the method described by Dengqiang Li et al. [23]. Acarbose, a definite α-glucosidase inhibition, was used as positive drug.

## 4. Conclusions

In summary, six new nortriterpenoids (**1**–**6**), together with nine known analogs (**7**–**15**), were isolated and identified from the fruiting bodies of *Ganoderma resinaceum*. The possible biogenetic pathway for C24 nortriterpenoids was proposed Compounds **3**, **4**, and **7**–**15** displayed no significant α-glucosidase inhibitory activity, however, this finding may support that the side chain of ganoderma triterpenoids is critical for α-glucosidase inhibitory activity, especially the presence of carboxyl acid group.

## Figures and Tables

**Table 1:** ^1^H-NMR (600 MHz, CD_3_OD) and ^13^C-NMR (150 MHz, CD_3_OD) spectroscopic data of **1**–**4**.

No.	1		2		3		4	
	δC mult.	δH mult. (*J* in Hz)	δC mult.	δH mult. (*J* in Hz)	δC mult.	δH mult. (*J* in Hz)	δC mult.	δH mult. (*J* in Hz)
**1**	36.0, CH2	2.20, dt (12.6, 3.0); 1.36, m	36.0, CH2	2.78, dt (13.8, 3.0); 1.02, m	34.9, CH2	2.75, dt (13.8, 3.6); 1.16, dt (13.8, 3.6)	34.9, CH2	2.80, m; 1.26, dt (13.5, 3.0)
**2**	28.3, CH2	1.60, m; 1.53, m	28.4, CH2	1.60, m; 1.68, m	28.5, CH2	1.66, m; 1.60, m	28.2, CH2	1.72, brd (13.2); 1.66, m
**3**	79.5, CH	3.08, dd (12.0, 4.2)	79.1, CH	3.17, dd (12.0, 4.8)	78.3, CH	3.14, dd (11.4, 4.8)	78.3, CH	3.21, dd (12.0, 4.2)
**4**	40.1, C		39.4, C		40.4, C		40.3, C	
**5**	51.7, CH	0.93, m	50.4, CH	0.96, d (13.2)	52.8, CH	1.50, d(1.8)	52.6, CH	1.60, d (14.4)
**6**	28.94, CH2	2.10, m; 1.61, m	28.0, CH2	2.20, dd (13.2, 9.0); 1.59, m	37.5, CH2	2.60, d (14.4); 2.40, dd (14.4, 1.8)	37.5, CH2	2.68, t (14.4); 2.49, d (14.4)
**7**	66.9, CH	4.54, t (9.6)	68.2, CH	4.85, t (9.0)	202.3, C		202.0, C	
**8**	136.8, C		158.2, C		147.3, C		147.8, C	
**9**	146.2, C		143.3, C		154.1, C		153.6, C	
**10**	40.2, C		39.8, C		41.9, C		41.9, C	
**11**	65.3, CH	4.48, d (7.2)	201.5, C		202.3, C		201.5, C	
**12**	42.0, CH2	2.51, dd (14.4, 7.2); 2.00, d (14.4)	51.3, CH2	2.89, d (16.2); 2.82, d (16.2)	50.2, CH2	2.90, d (15.6); 2.78, d (15.6)	50.5, CH2	3.05, d (15.6); 2.65, d (15.6)
**13**	43.8 C		46.6, C		45.3, C		45.7, C	
**14**	61.0, C		60.3, C		58.1, C		58.7, C	
**15**	220.2, C		218.6, C		210.2, C		210.8, C	
**16**	37.3, CH2	2.82, dd (19.8, 8.4); 2.45, dd (19.8, 9.0)	40.0, CH2	2.63, dd (19.8, 9.0); 2.06, dd (19.8, 9.6)	38.5, CH2	2.60, dd (18.6, 9.0); 1.69, dd (18.6, 8.4)	41.3, CH2	2.82, m; 1.87, dd (18.0, 7.8)
**17**	54.8, CH	3.22, overlapped	48.9, CH	2.28, dd (18.6, 9.0)	47.9, CH	2.28, dd (18.6, 9.6)	46.2, CH	2.34, m
**18**	19.7, CH3	0.79, s	18.1, CH3	1.03, s	16.9, CH3	0.80, s	16.7, CH3	0.83, s
**19**	22.2, CH3	1.21, s	19.0, CH3	1.25, s	18.2, CH3	1.26, s	18.3, CH3	1.30, s
**20**	209.3, C		69.9, CH	3.79, m	69.9, CH	3.65, m	32.9, CH2	2.13, m
**21**	31.8, CH3	2.13, s	23.8, CH3	1.20, d (6.0)	23.7, CH3	1.10, d (6.0)	20.5, CH3	0.99, t (6.0)
**28**	28.89, CH3	0.90, s	28.8, CH3	1.04, s	28.2, CH3	0.94, s	28.5, CH3	1.00, s
**29**	16.2, CH3	0.77, s	16.4, CH3	0.86, s	16.8, CH3	1.13, s	16.3, CH3	0.88, s
**30**	23.5, CH3	1.08, s	24.6, CH3	1.42, s	16.3, CH3	0.82, s	22.0, CH3	1.57, s

**Table 2:** ^1^H-NMR (600 MHz) and ^13^C-NMR (150 MHz) spectroscopic data of **5** and **6**.

No.	5a		6b	
	δC mult.	δH mult. (*J* in Hz)	δC mult.	δH mult. (*J* in Hz)
**1**	34.7, CH2	2.94, dt (13.2, 3.6); 1.07, m	36.0, CH2	2.71, dt (13.2, 3.6); 0.95, m
**2**	27.7, CH2	1.70, m	28.4, CH2	1.58, m; 1.53, m
**3**	78.2, CH	3.26, dd (12.0, 4.8)	79.0, CH	3.08, dd (12.0, 4.8)
**4**	38.7, C		39.7, C	
**5**	49.3, CH	0.98, d (13.2)	50.4, CH	0.87, d (13.8)
**6**	26.1, CH2	2.20, dd (13.2, 7.2); 1.64, m	28.1, CH2	2.11, m; 1.49, m
**7**	67.1, CH	4.84, d (4.2)	68.1, CH	4.78, t (3.0)
**8**	158.4, C		159.0, C	
**9**	142.2, C		144.2, C	
**10**	39.2, C		40.0, C	
**11**	197.6, C		200.6, C	
**12**	44.2, CH2	2.98, d (16.2); 2.58, d (16.2)	51.6, CH2	2.85, d (16.8); 2.55, d (16.8)
**13**	51.3, C		46.7, C	
**14**	58.2, C		60.2, C	
**15**	211.0, C		218.9, C	
**16**	123.5, CH	5.84, s	41.6, CH2	2.68, d (13.2); 2.10, m
**17**	185.6, C		43.7, CH	2.15, m
**18**	30.6, CH3	1.24, s	16.4, CH3	0.77, s
**19**	18.6, CH3	1.23, s	19.0, CH3	1.15, s
**20**	36.3, CH	2.65, m	39.8, CH	1.58, m
**21**	16.5, CH3	1.18, d (7.2)	17.5, CH3	1.00, d (6.6)
**22**	65.6, CH2	3.79, m	67.6, CH2	3.45, dd (11.4, 3.3); 3.32, dd (11.4, 6.0)
**28**	28.2, CH3	1.07, s	28.8, CH3	0.95, s
**29**	15.6, CH3	0.88, s	18.0, CH3	0.91, s
**30**	33.5, CH3	1.58, s	25.0, CH3	1.30, s

^a^ measured in CDCl_3_; ^b^ measured in CD_3_OD.

## Supplementary Materials

The following are available online: Spectrum copies of Mass, NMR, and IR.
