# Supplementary material for: Nortriterpenoids from the Fruiting Bodies of the Mushroom Ganoderma resinaceum
Source: Molecules. 2017 Jun 28;22(7):1073. doi: 10.3390/molecules22071073 (PMC6152414; doi:10.3390/molecules22071073)
Supplement: Supplementary file 1 [file molecules-22-01073-s001.zip › molecules-203943-supplementary/molecules-203943-supplementary.pdf]

Supplementary data

Nortriterpenoids from the fruiting bodies of the mushroom *Ganoderma resinaceum*

Xian-Qiang Chen [1], Ling-Xiao Chen [1], Jing Zhao [1,*], Yu-Ping Tang [2] and Shao-Ping Li [1,*]

[1] State Key Laboratory of Quality Research in Chinese Medicine, Institute of Chinese Medical Sciences, University of Macau, Macau, China; yb27528@umac.mo (X.-Q.C.); yb47517@umac.mo (L.-X.C.)
[2] Jiangsu Collaborative Innovation Center of Chinese Medicinal Resources Industrialization, Jiangsu Key Laboratory for High Technology Research of TCM Formulae, and National and Local Collaborative Engineering Center of Chinese Medicinal Resources Industrialization and Formulae Innovative Medicine, Nanjing University of Chinese Medicine, Jiangsu, China
* Correspondence: zhaojing.cpu@163.com (J.Z.); spli@umac.mo (S.-P.L.); Tel.: +853-88224692; Fax: +853-28841358

List of contents

Fig. S1 Separation flow chart of *Ganoderma resinaceum*

Fig. S2 ^{1}H NMR (600 MHz, CD_3OD) spectrum of lucidone I (**1**)

Fig. S3 ^{13}C NMR (150 MHz, CD_3OD) spectrum of lucidone I (**1**)

Fig. S4 HSQC (600 MHz, CD_3OD) spectrum of lucidone I (**1**)

Fig. S5 HMBC (600 MHz, CD_3OD) spectrum of lucidone I (**1**)

Fig. S6 ^{1}H-^{1}H COSY (600 MHz, CD_3OD) spectrum of lucidone I (**1**)

Fig. S7 ROESY (600 MHz, CD_3OD) spectrum of lucidone I (**1**)

Fig. S8 IR spectrum of lucidone I (**1**)

Fig. S9 HRESIMS spectrum of lucidone I (**1**)

Fig. S10 ^{1}H NMR (600 MHz, CD_3OD) spectrum of lucidone J (**2**)

Fig. S11 ^{13}C NMR (150 MHz, CD_3OD) spectrum of lucidone J (**2**)

Fig. S12 HSQC (600 MHz, CD_3OD) spectrum of lucidone J (**2**)

Fig. S13 HMBC (600 MHz, CD_3OD) spectrum of lucidone J (**2**)

Fig. S14 ^{1}H-^{1}H COSY (600 MHz, CD_3OD) spectrum of lucidone J (**2**)

Fig. S15 ROESY (600 MHz, CD_3OD) spectrum of lucidone J (**2**)

Fig. S16 IR spectrum of lucidone J (**2**)

Fig. S17 HRESIMS spectrum of lucidone J (**2**)

Fig. S18 ^{1}H NMR (600 MHz, CD_3OD) spectrum of lucidone K (**3**)

Fig. S19 ^{13}C NMR (150 MHz, CD_3OD) spectrum of lucidone K (**3**)

Fig. S20 HSQC (600 MHz, CD_3OD) spectrum of lucidone K (**3**)

Fig. S21 HMBC (600 MHz, CD_3OD) spectrum of lucidone K (**3**)

Fig. S22 ^{1}H-^{1}H COSY (600 MHz, CD_3OD) spectrum of lucidone K (**3**)

Fig. S23 ROESY (600 MHz, CD_3OD) spectrum of lucidone K (**3**)

Fig. S24 IR spectrum of lucidone K (**3**)

Fig. S25 HRESIMS spectrum of lucidone K (**3**)

Fig. S26 ^{1}H NMR (600 MHz, CD_3OD) spectrum of lucidone L (**4**)

Fig. S27 ^{13}C NMR (150 MHz, CD_3OD) spectrum of lucidone L (**4**)

Fig. S28 HSQC (600 MHz, CD_3OD) spectrum of lucidone L (**4**)

Fig. S29 HMBC (600 MHz, CD_3OD) spectrum of Lucidone L (**4**)

Fig. S30 ^{1}H-^{1}H COSY (600 MHz, CD_3OD) spectrum of lucidone L (**4**)

Fig. S31 ROESY (600 MHz, CD_3OD) spectrum of lucidone L (**4**)

Fig. S32 IR spectrum of lucidone L (**4**)

Fig. S33 HRESIMS spectrum of lucidone L (**4**)

Fig. S34 ^{1}H NMR (600 MHz, $CDCl_3$) spectrum of ganosineniol B (**5**)

Fig. S35 ^{13}C NMR (150 MHz, $CDCl_3$) spectrum of ganosineniol B (**5**)

Fig. S36 DEPT135 NMR (150 MHz, $CDCl_3$) spectrum of ganosineniol B (**5**)

Fig. S37 HMBC (600 MHz, $CDCl_3$) spectrum of ganosineniol B (**5**)

Fig. S38 ^{1}H-^{1}H COSY (600 MHz, $CDCl_3$) spectrum of ganosineniol B (**5**)

Fig. S39 ROESY (600 MHz, $CDCl_3$) spectrum of ganosineniol B (**5**)

Fig. S40 IR spectrum of ganosineniol B (**5**)

Fig. S41 HRESIMS spectrum of ganosineniol B (**5**)

Fig. S42 ^{1}H NMR (600 MHz, CD_3OD) spectrum of ganosineniol C (**6**)

Fig. S43 ^{13}C NMR (150 MHz, CD_3OD) spectrum of ganosineniol C (**6**)

Fig. S44 HSQC (600 MHz, CD_3OD) spectrum of ganosineniol C (**6**)

Fig. S45 HMBC (600 MHz, CD_3OD) spectrum of ganosineniol C (**6**)

Fig. S46 ^{1}H-^{1}H COSY (600 MHz, CD_3OD) spectrum of ganosineniol C (**6**)

Fig. S47 ROESY (600 MHz, CD_3OD) spectrum of ganosineniol C (**6**)

Fig. S48 IR spectrum of ganosineniol C (**6**)

Fig. S49 HRESIMS spectrum of ganosineniol C (**6**)

Table S1 Inhibition rate of nortriterpenoids from *Ganoderma* at the concentration of 3 mM

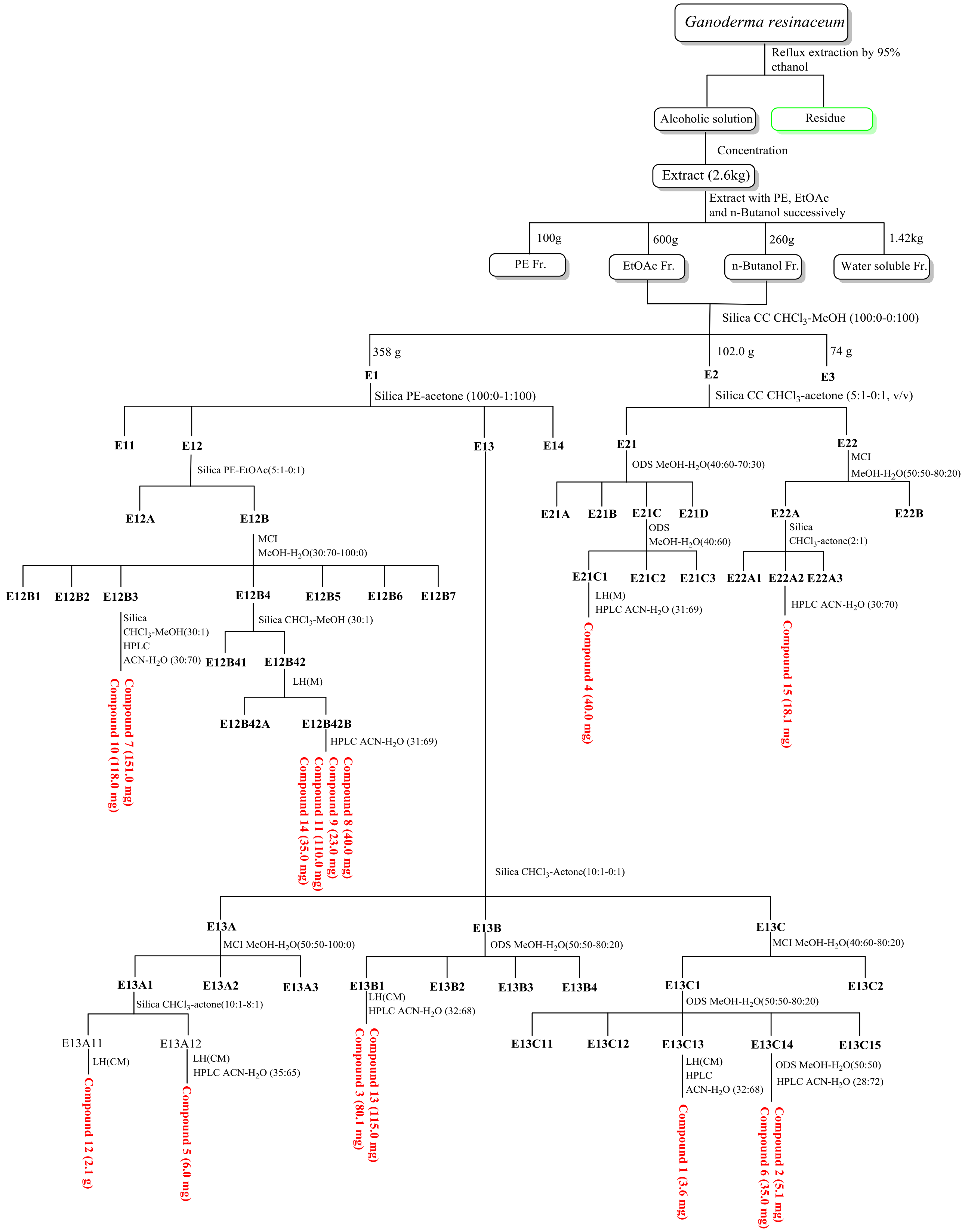

Fig. S1 Separation flow chart of *Ganoderma resinaceum*

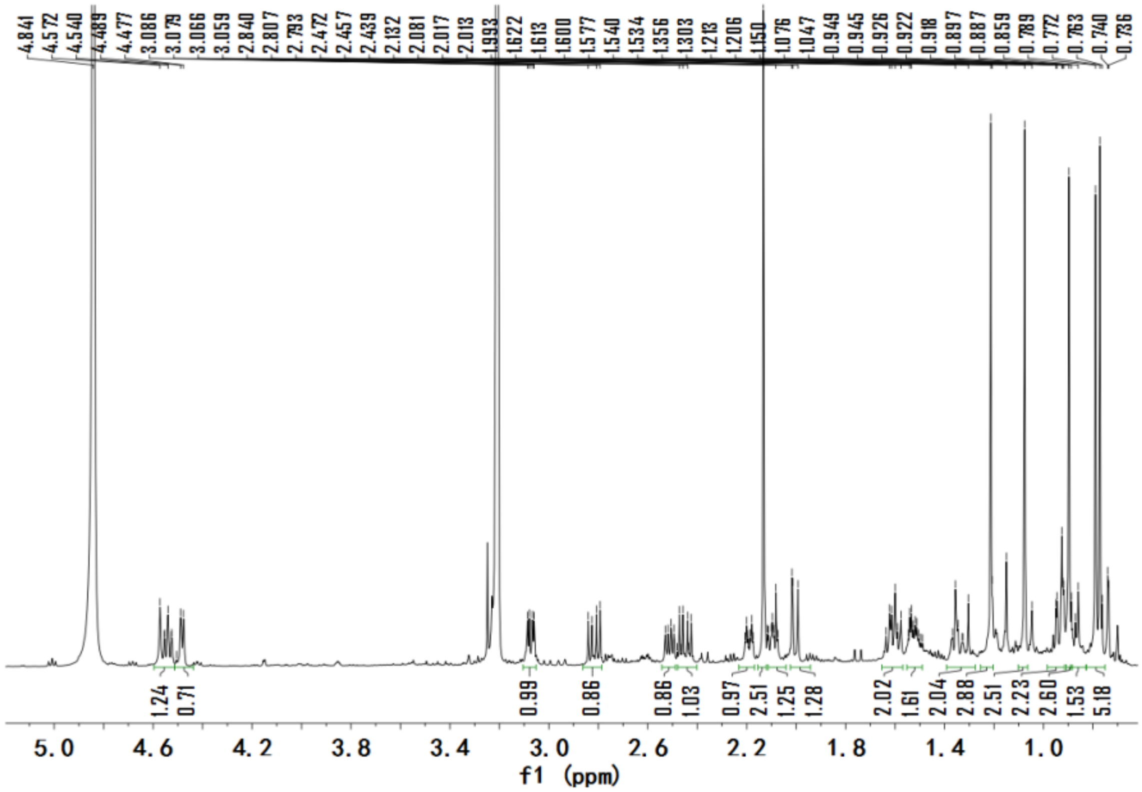

Fig. S2 ^{1}H NMR (600 MHz, CD_3OD) spectrum of lucidone I (**1**)

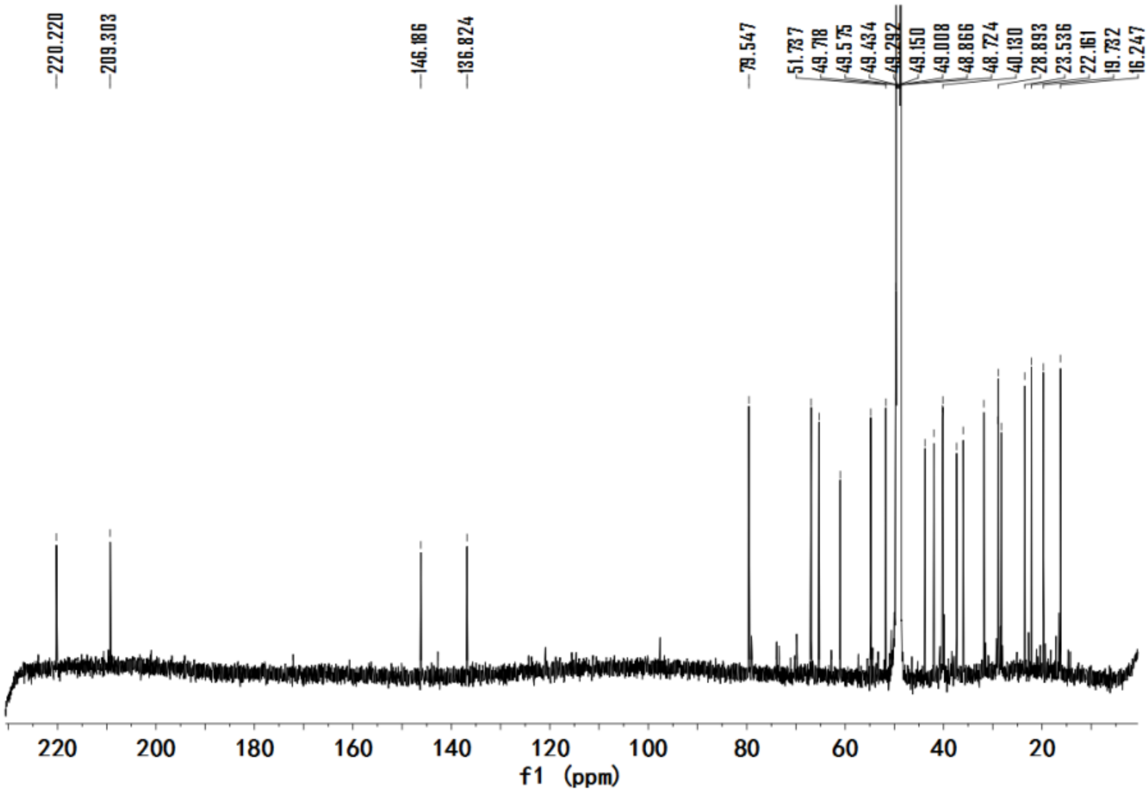

Fig. S3 ^{13}C NMR (150 MHz, CD_3OD) spectrum of lucidone I (**1**)

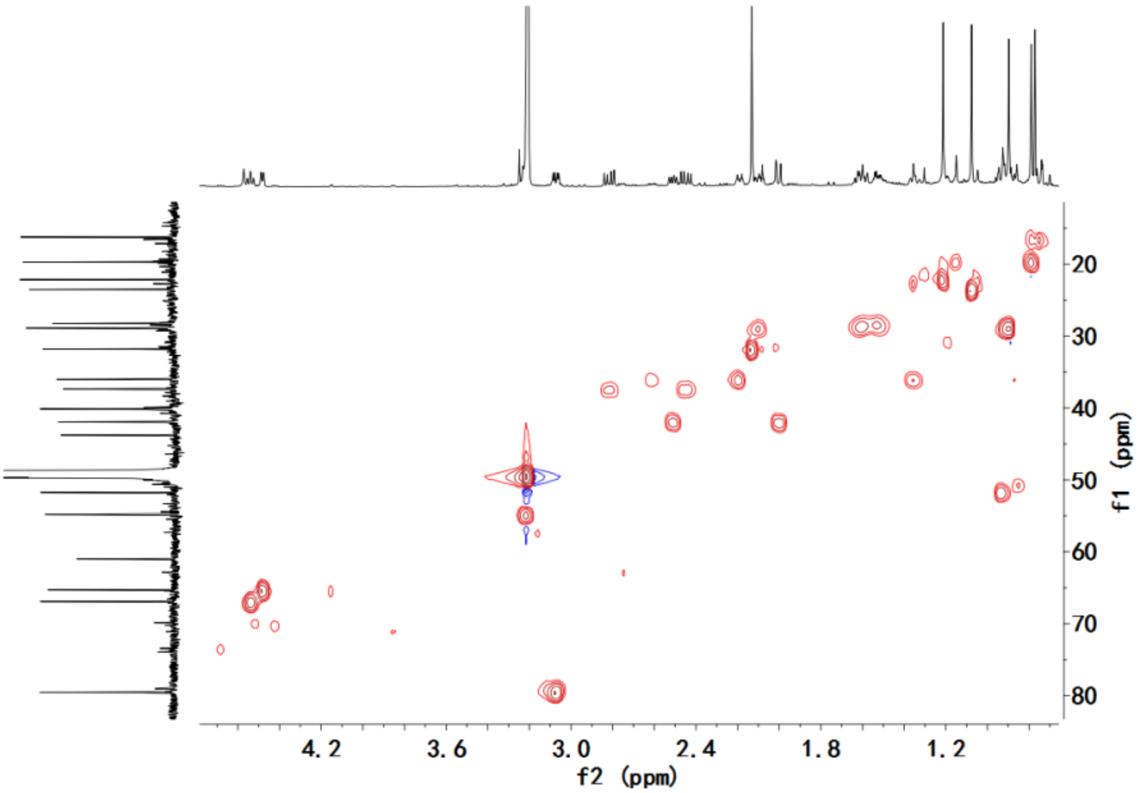

Fig. S4 HSQC (600 MHz, CD3OD) spectrum of lucidone I (**1**)

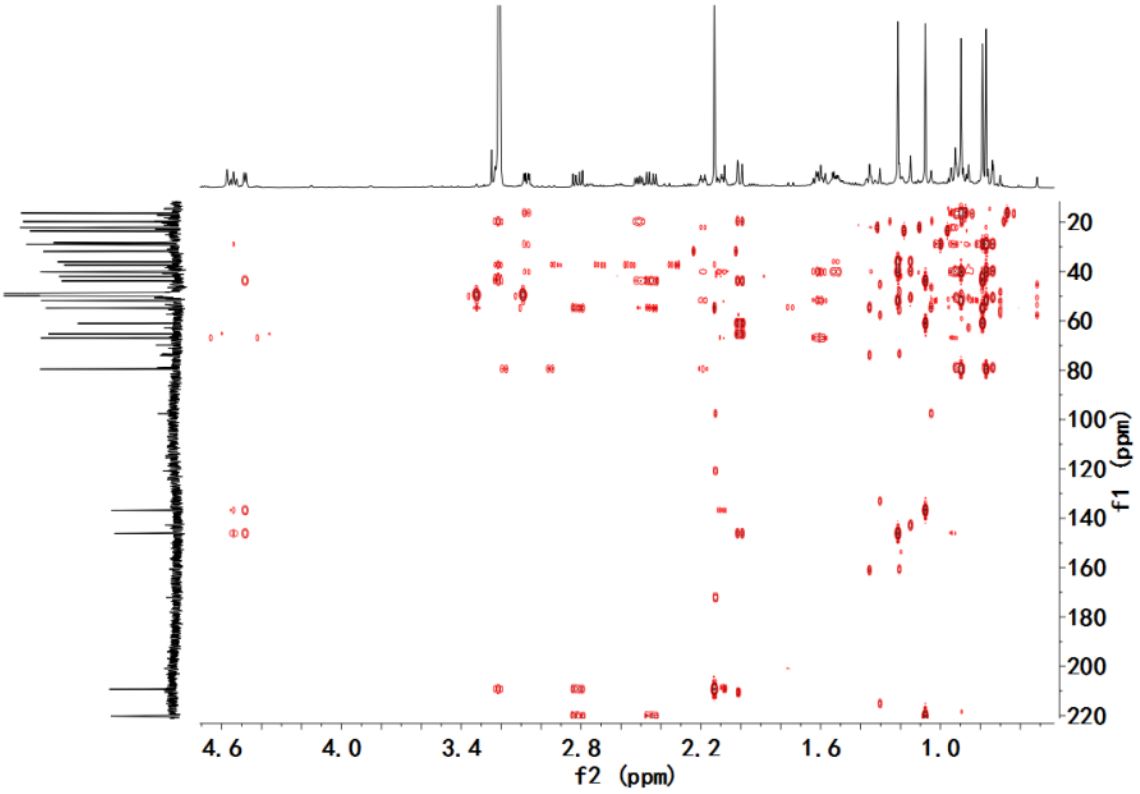

Fig. S5 HMBC (600 MHz, CD3OD) spectrum of lucidone I (**1**)

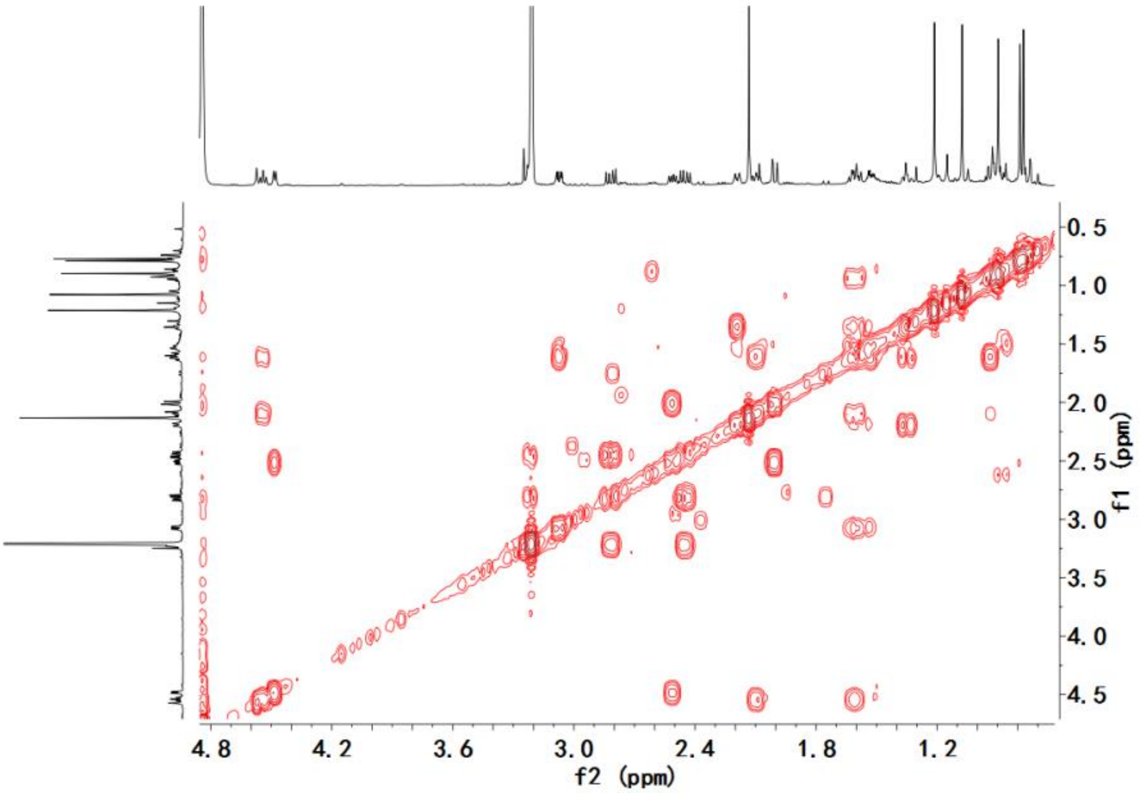

Fig. S6 ^{1}H-^{1}H COSY (600 MHz, CD_3OD) spectrum of lucidone I (**1**)

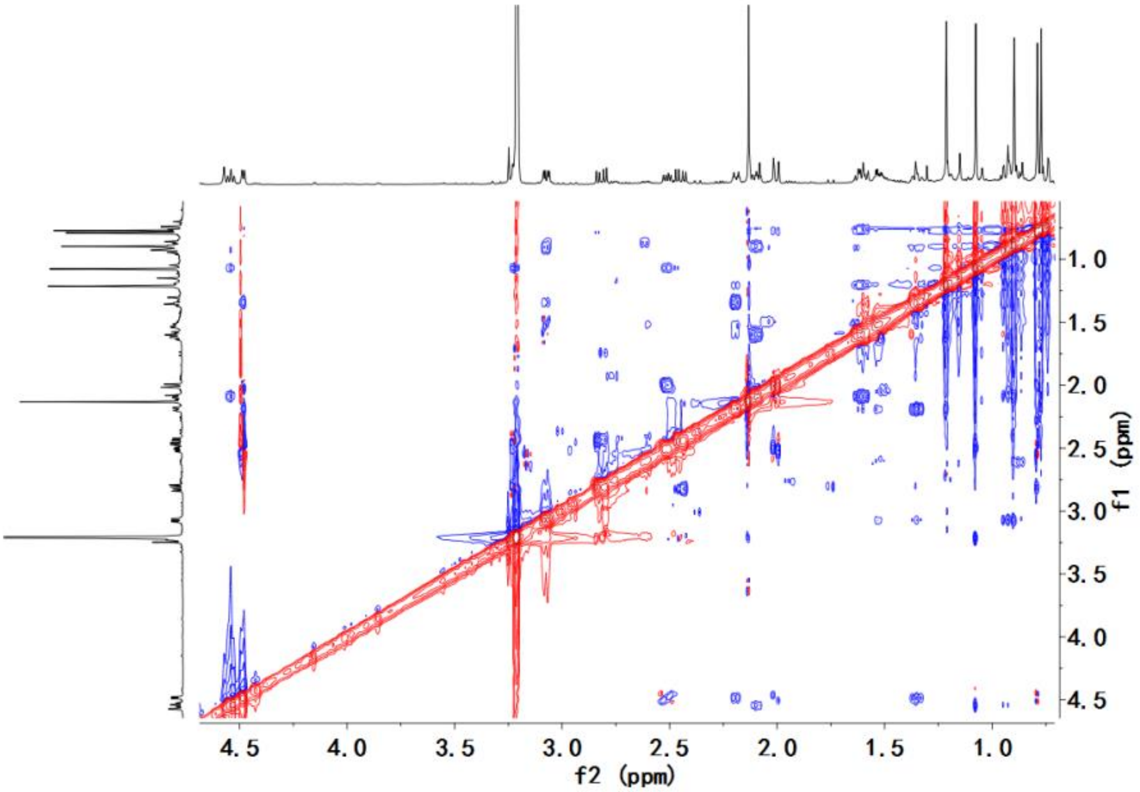

Fig. S7 ROESY (600 MHz, CD_3OD) spectrum of lucidone I (**1**)

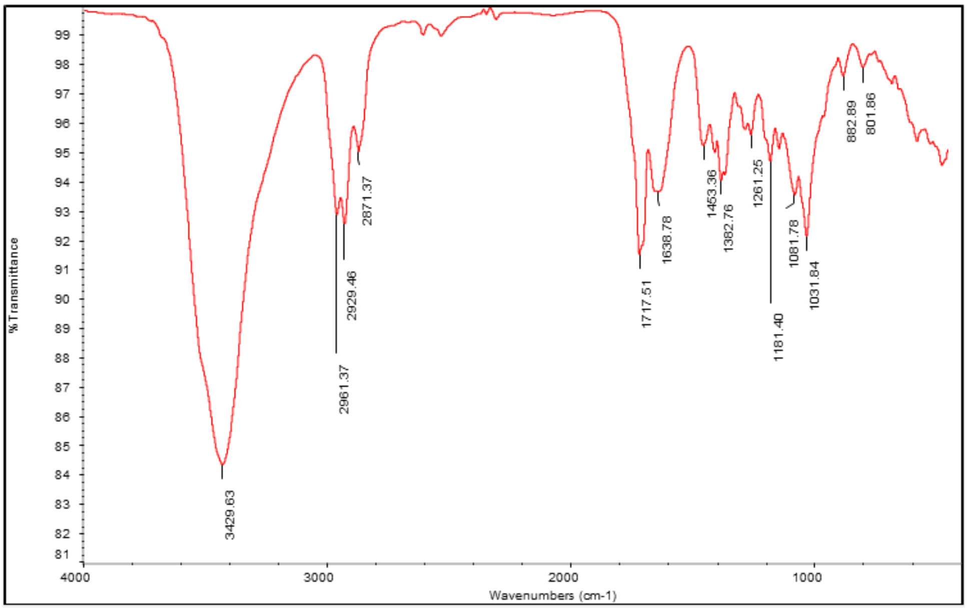

Fig. S8 IR spectrum of lucidone I (**1**)

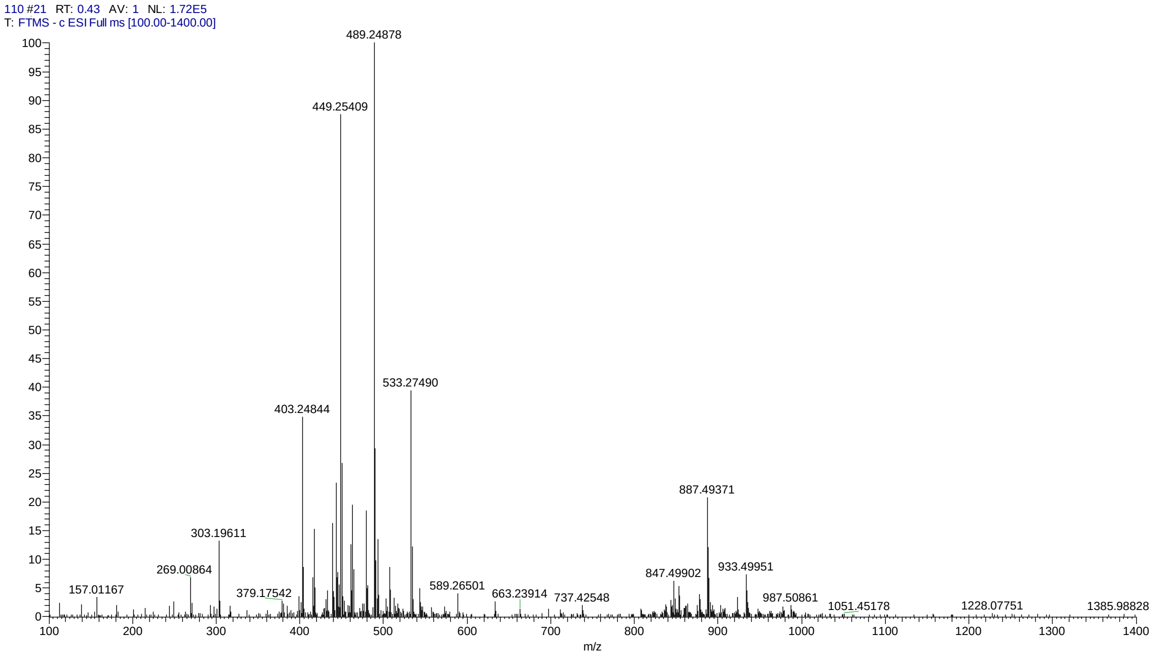

Fig. S9 HRESIMS spectrum of lucidone I (**1**)

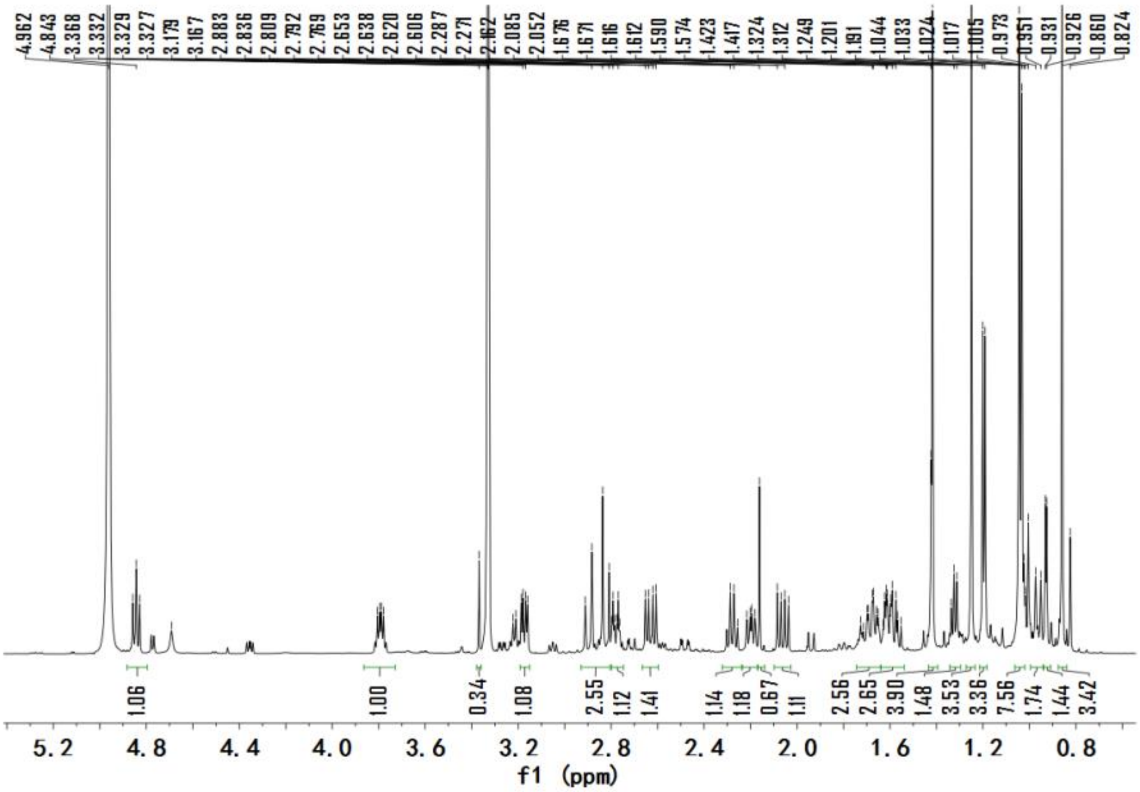

Fig. S10 ^{1}H NMR (600 MHz, CD_3OD) spectrum of lucidone J (**2**)

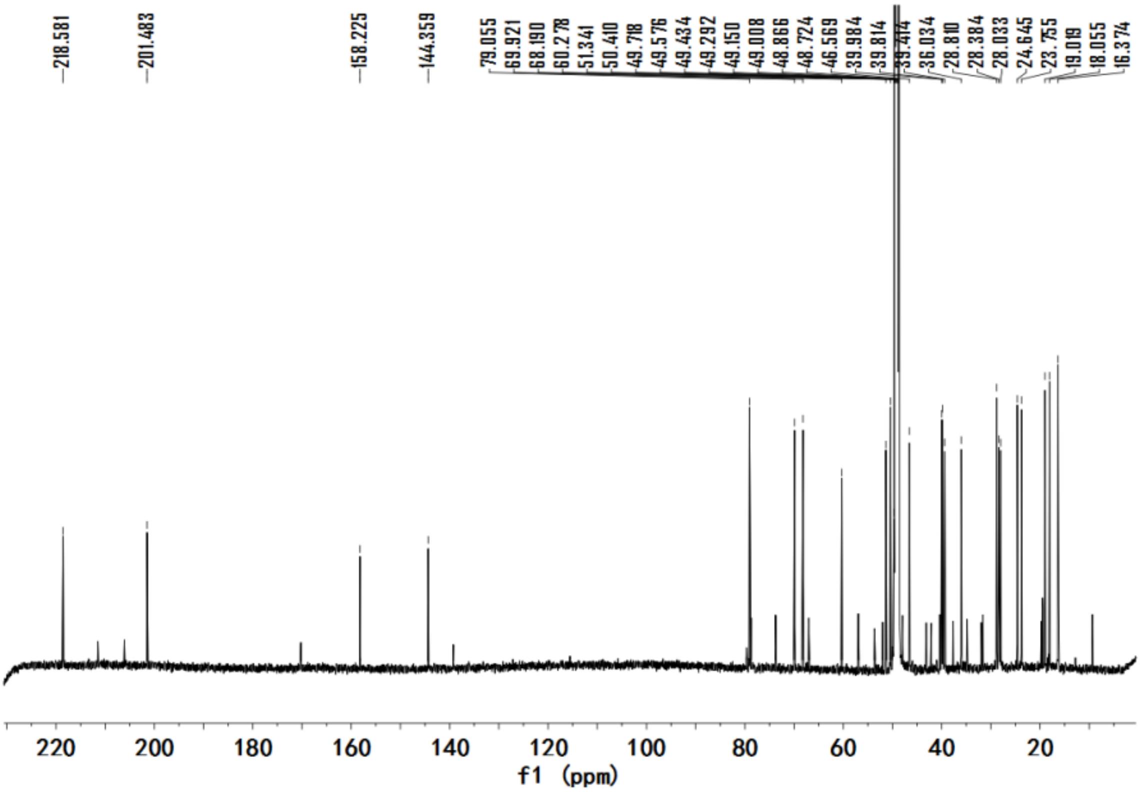

Fig. S11 ^{13}C NMR (150 MHz, CD_3OD) spectrum of lucidone J (**2**)

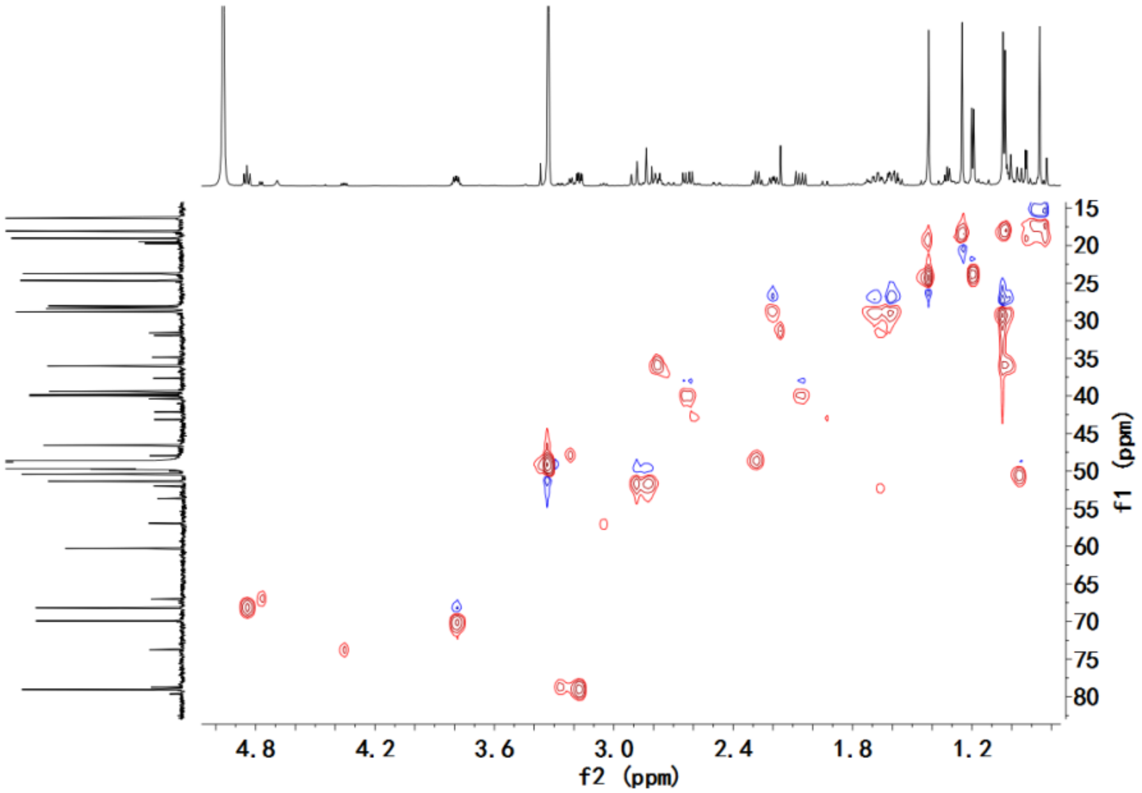

Fig. S12 HSQC (600 MHz, CD_3OD) spectrum of lucidone J (**2**)

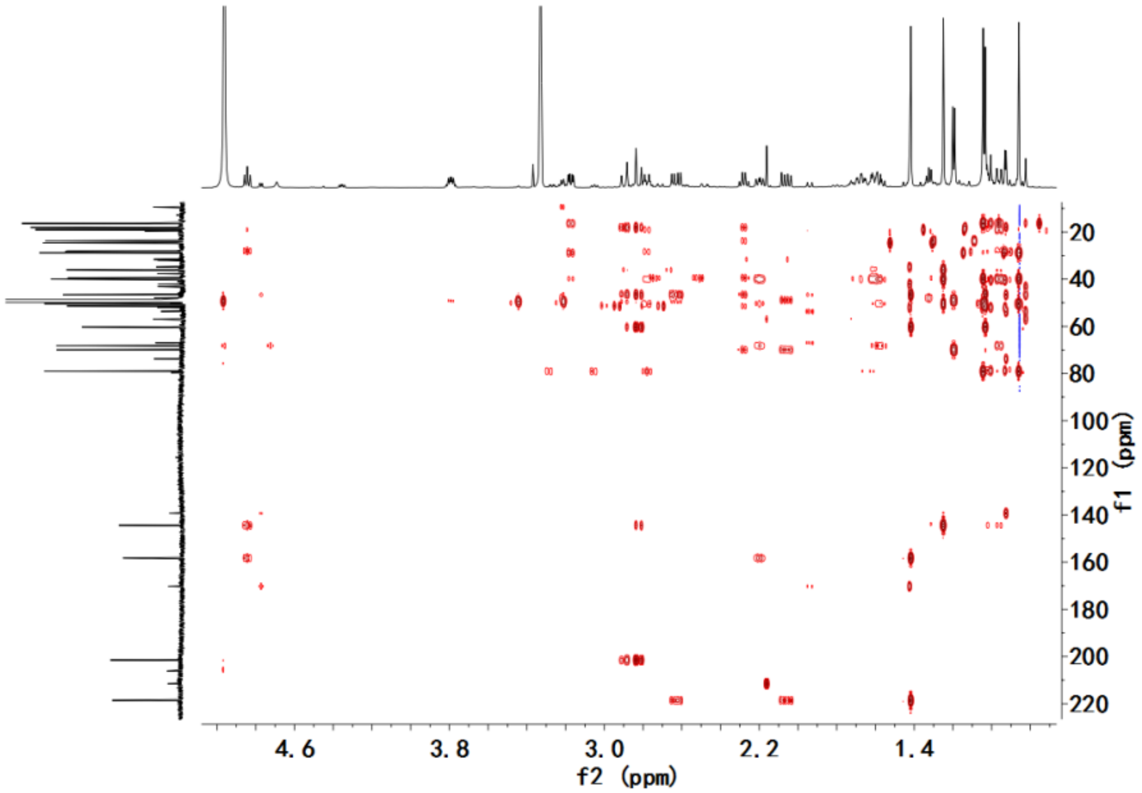

Fig. S13 HMBC (600 MHz, CD_3OD) spectrum of lucidone J (**2**)

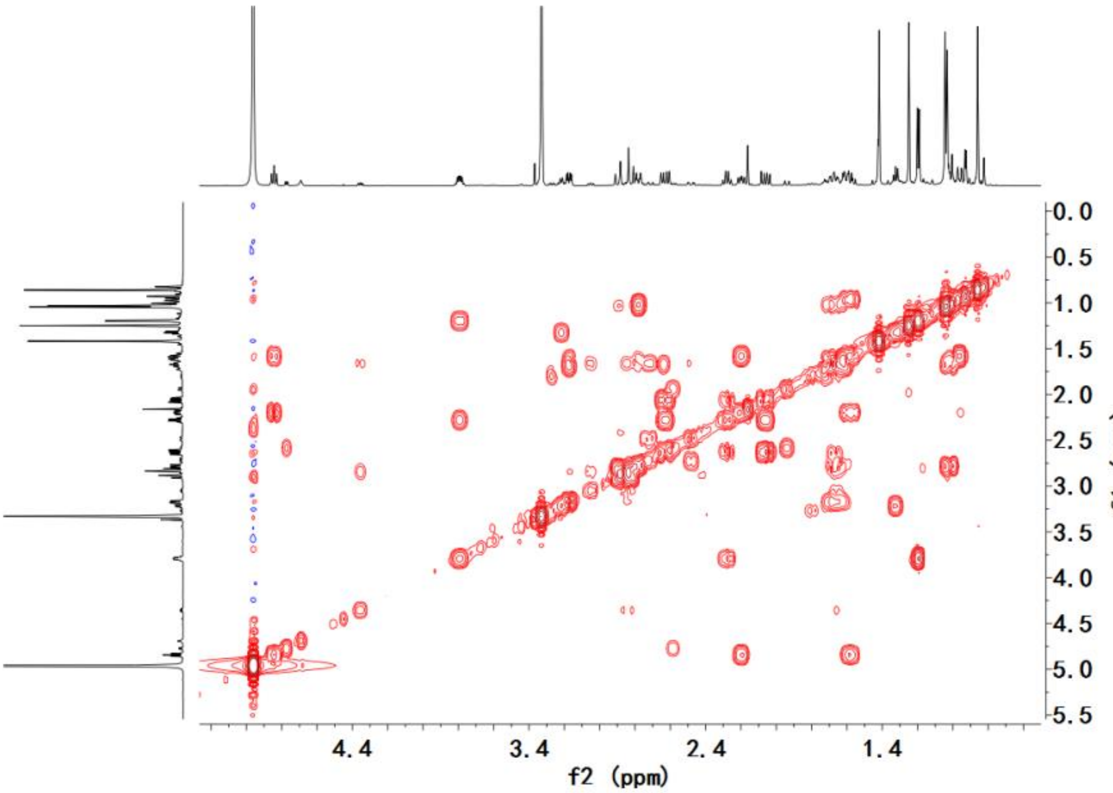

Fig. S14 ^{1}H-^{1}H COSY (600 MHz, CD_3OD) spectrum of lucidone J (**2**)

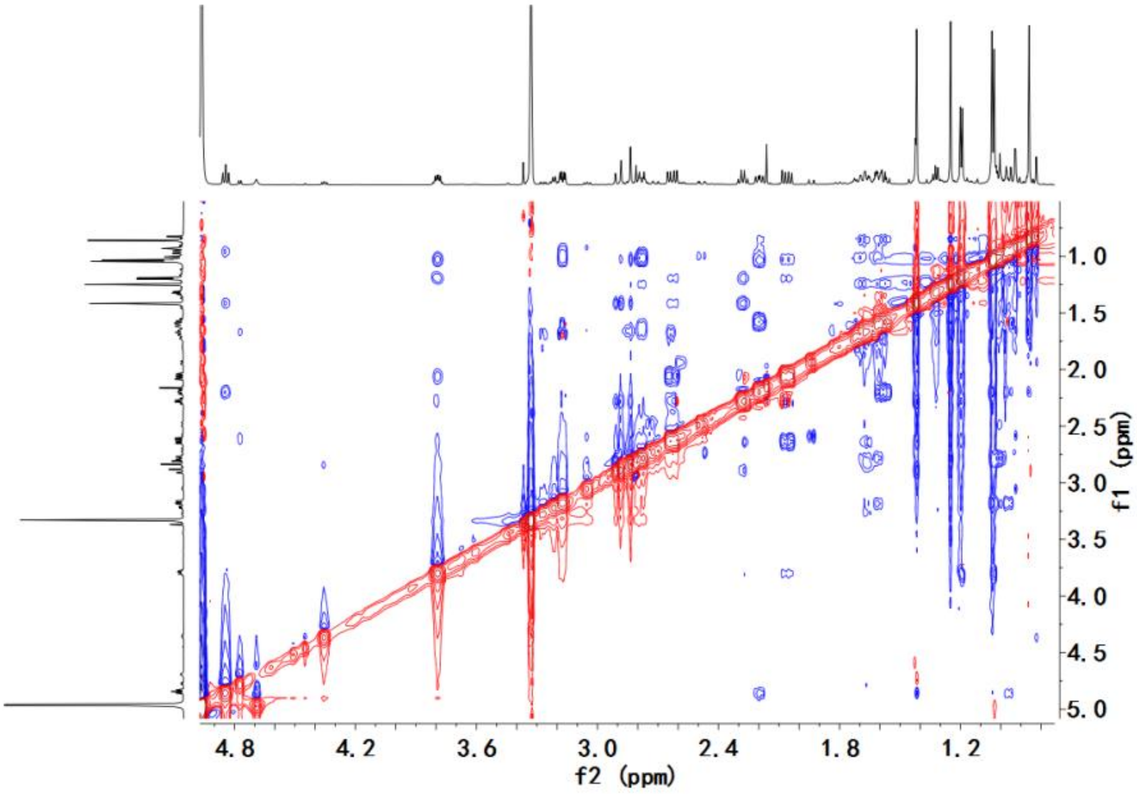

Fig. S15 ROESY (600 MHz, CD_3OD) spectrum of lucidone J (**2**)

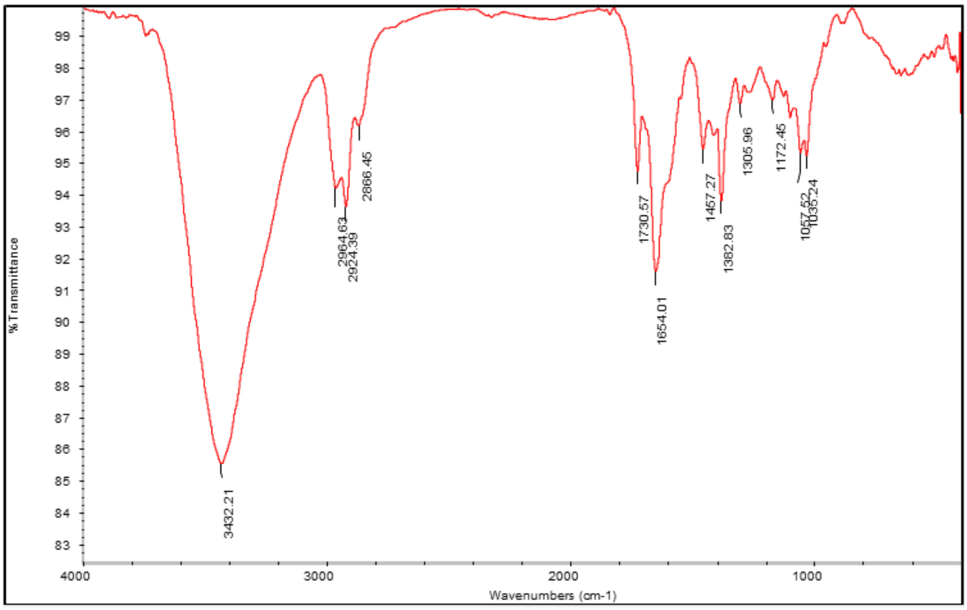

Fig. S16 IR spectrum of lucidone J (**2**)

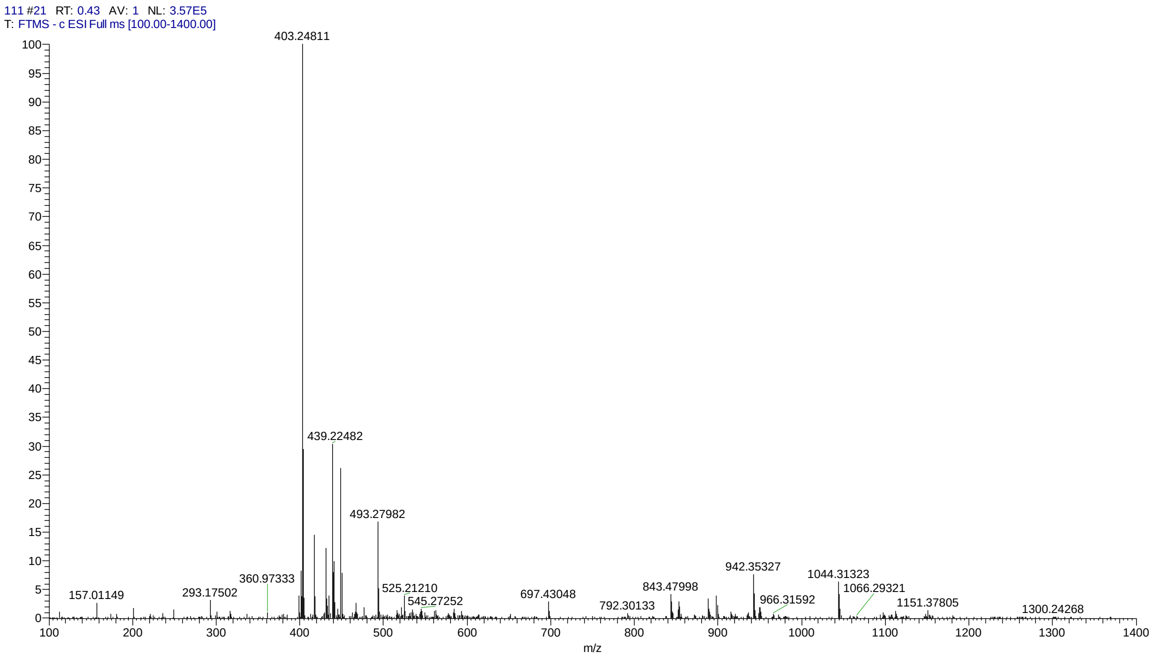

Fig. S17 HRESIMS spectrum of lucidone J (**2**)

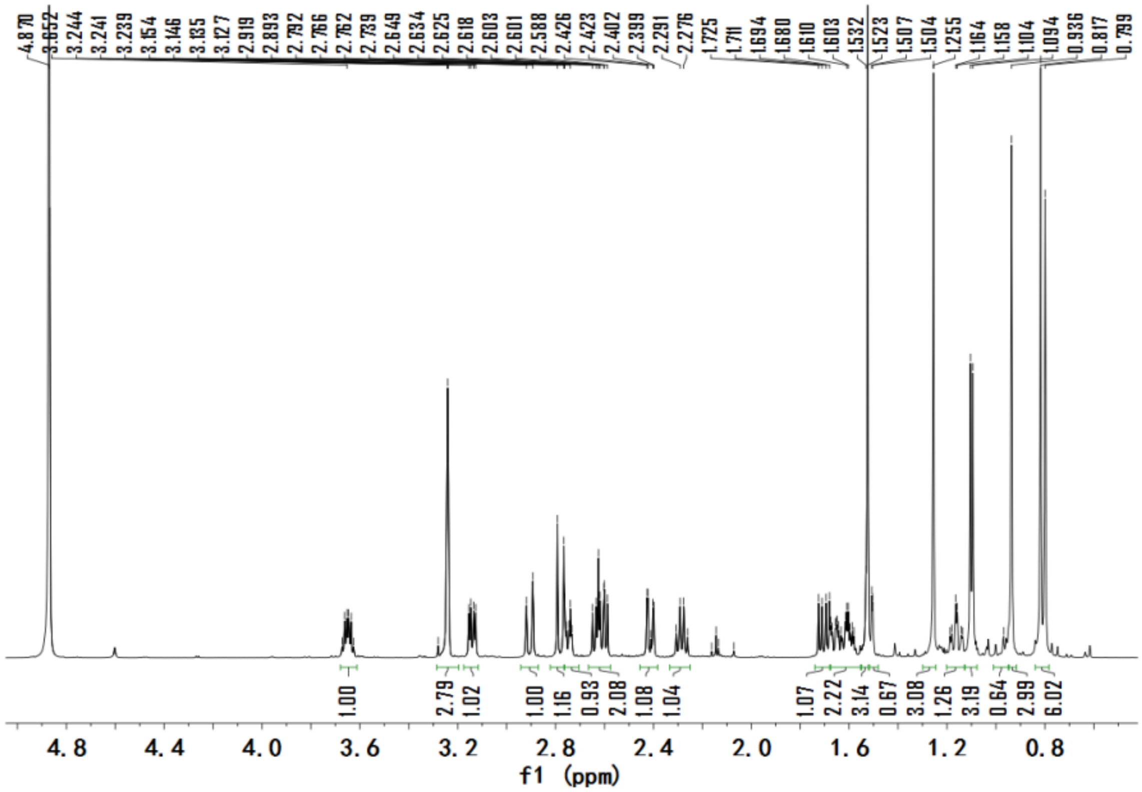

Fig. S18 ^{1}H NMR (600 MHz, CD_3OD) spectrum of lucidone K (**3**)

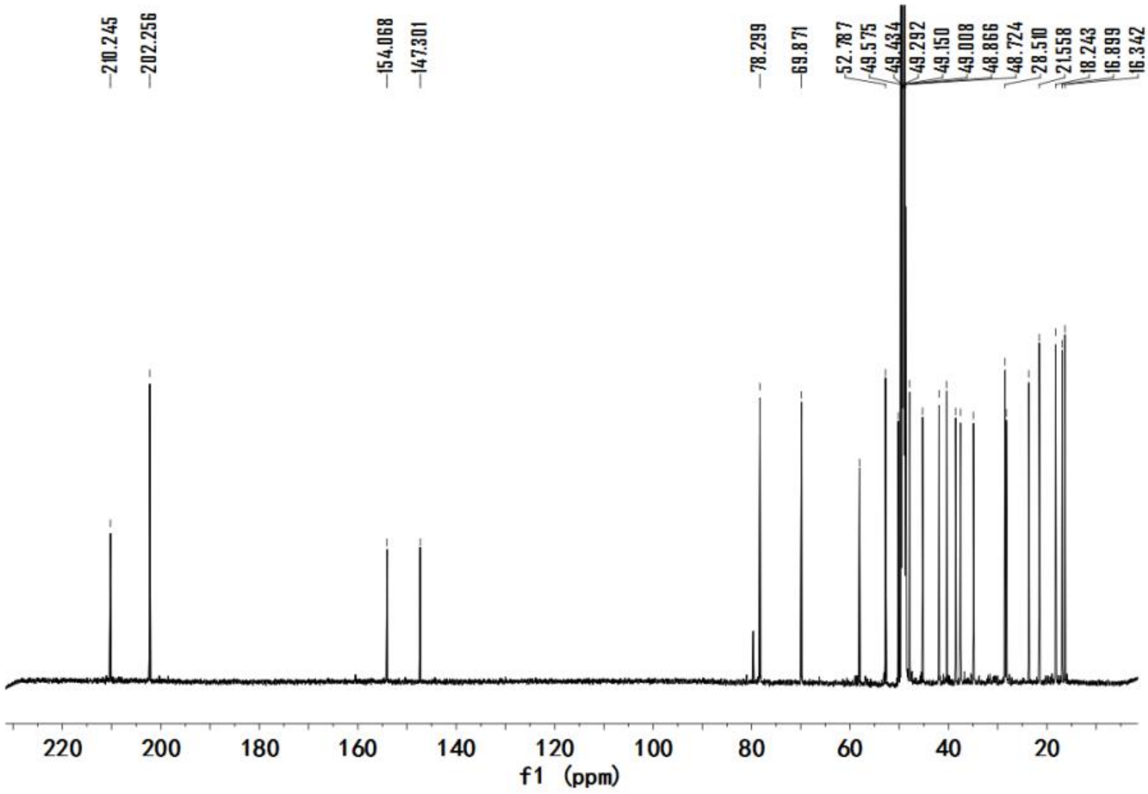

Fig. S19 ^{13}C NMR (150 MHz, CD_3OD) spectrum of lucidone K (**3**)

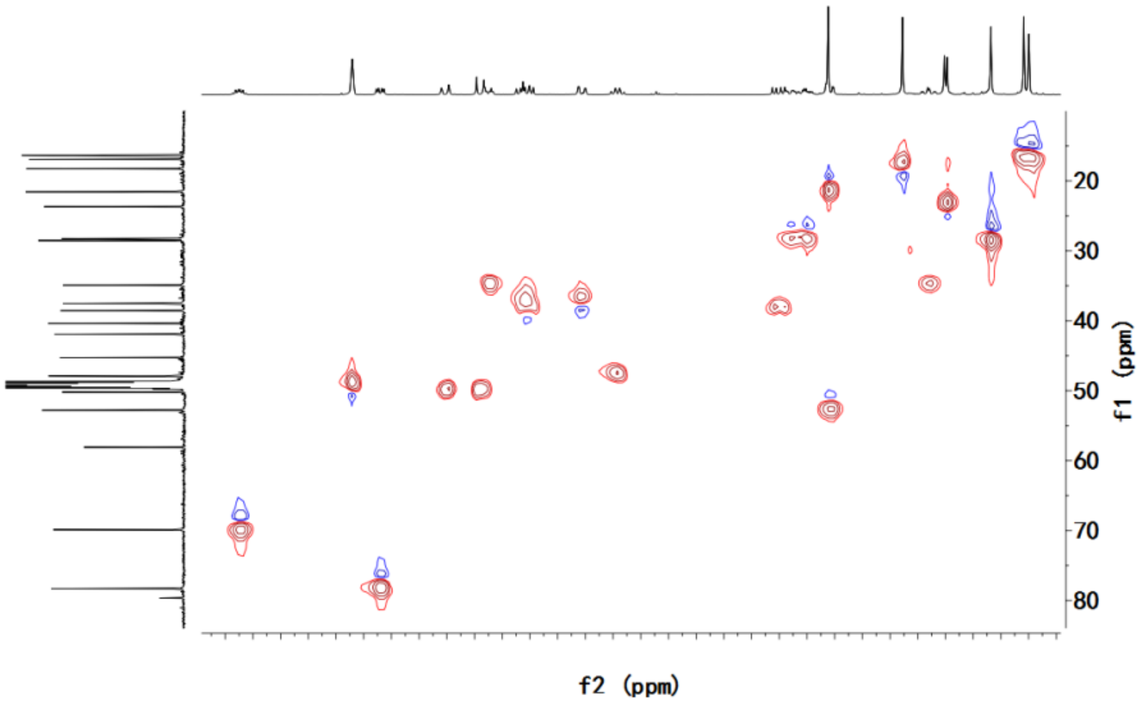

Fig. S20 HSQC (600 MHz, CD_3OD) spectrum of lucidone K (**3**)

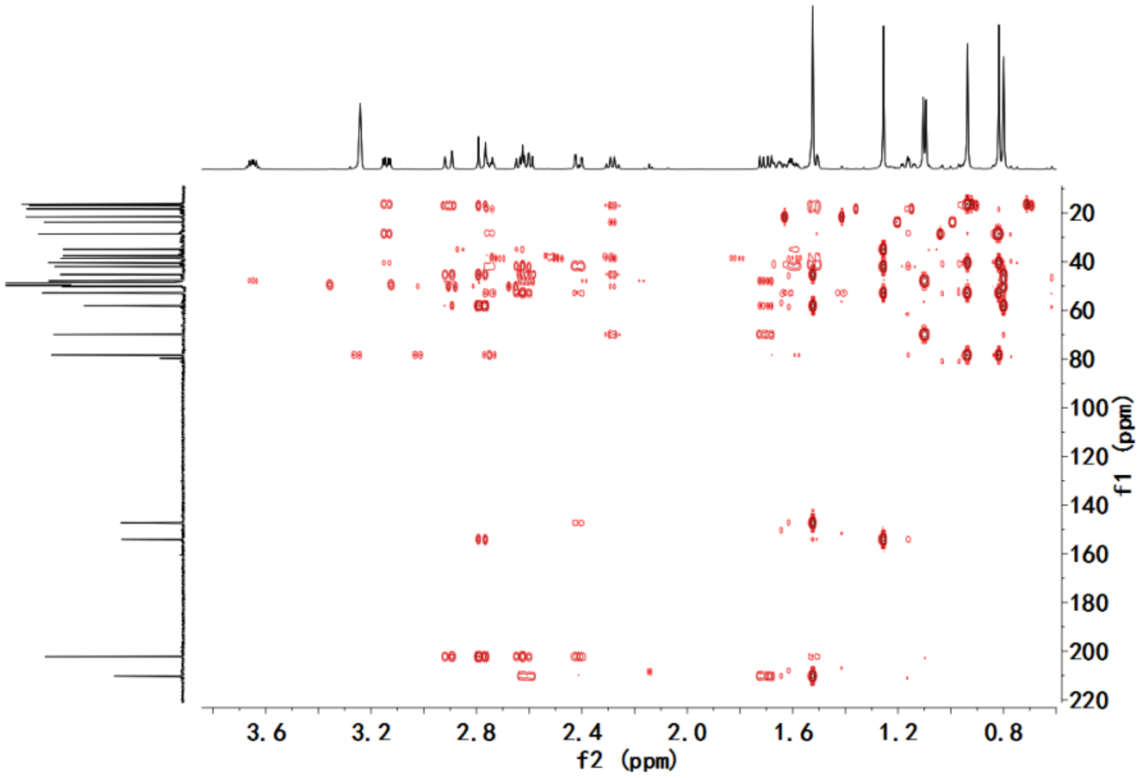

Fig. S21 HMBC (600 MHz, CD_3OD) spectrum of lucidone K (**3**)

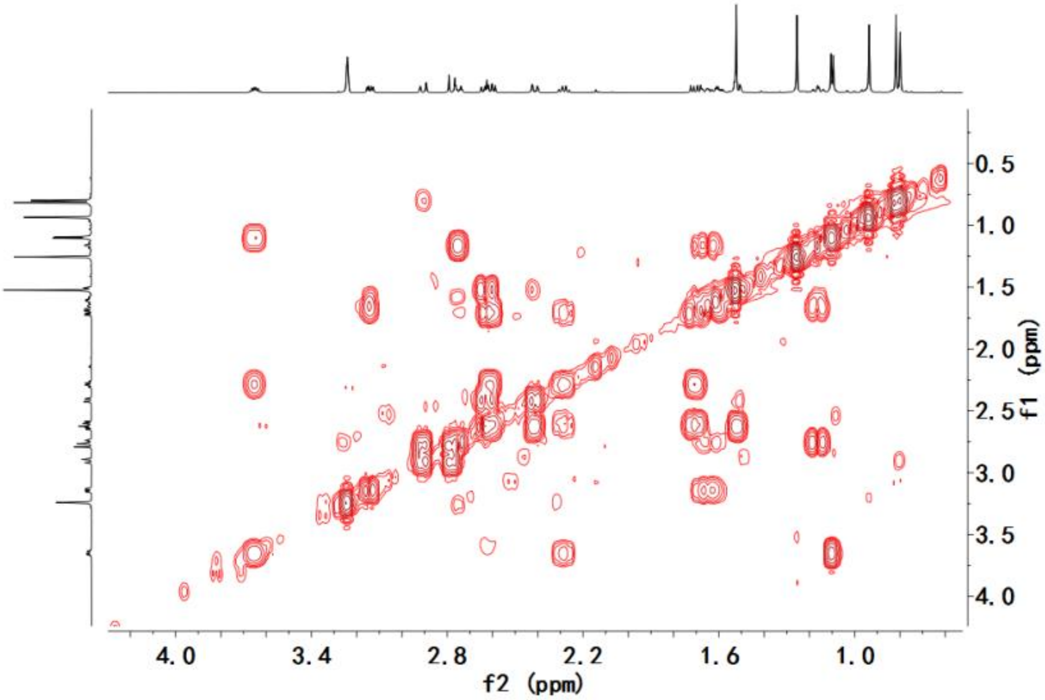

Fig. S22 ^{1}H-^{1}H COSY (600 MHz, CD_3OD) spectrum of lucidone K (**3**)

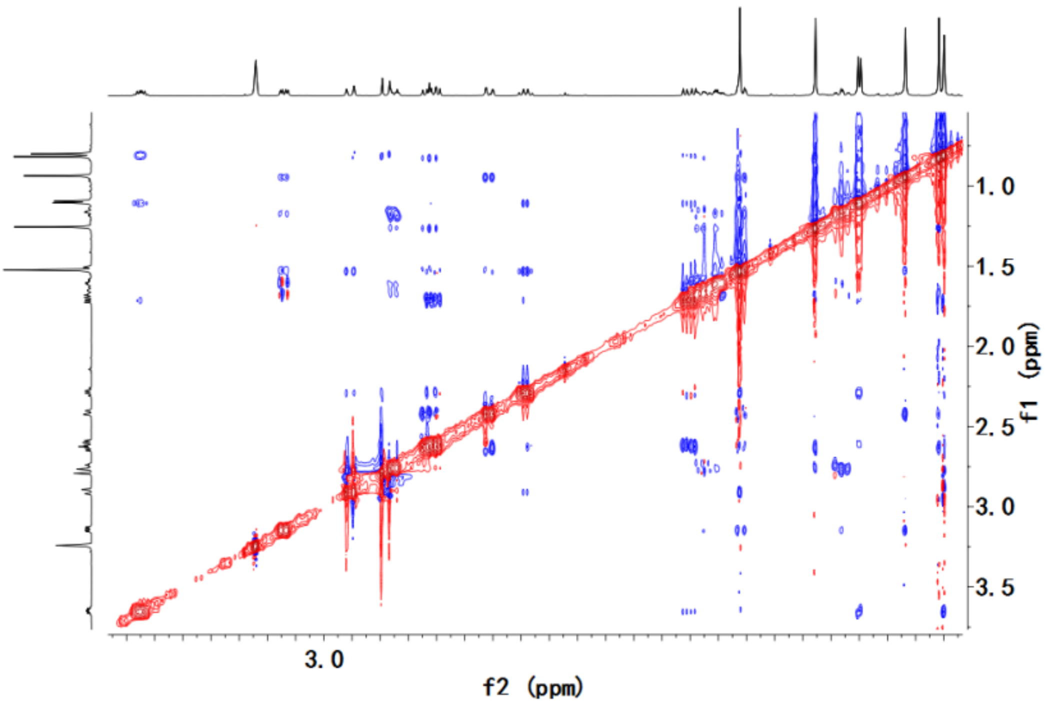

Fig. S23 ROESY (600 MHz, CD_3OD) spectrum of lucidone K (**3**)

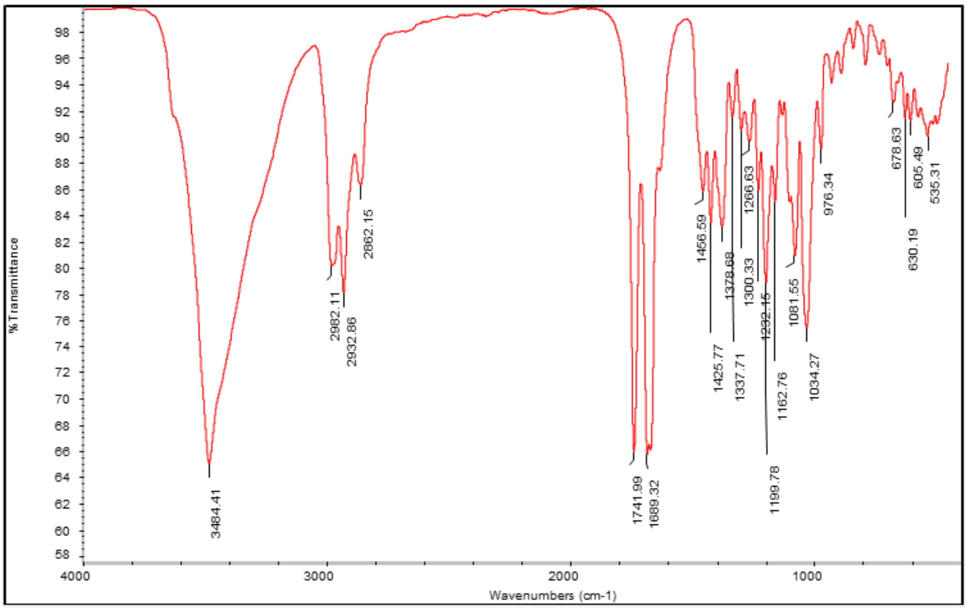

Fig. S24 IR spectrum of lucidone K (**3**)

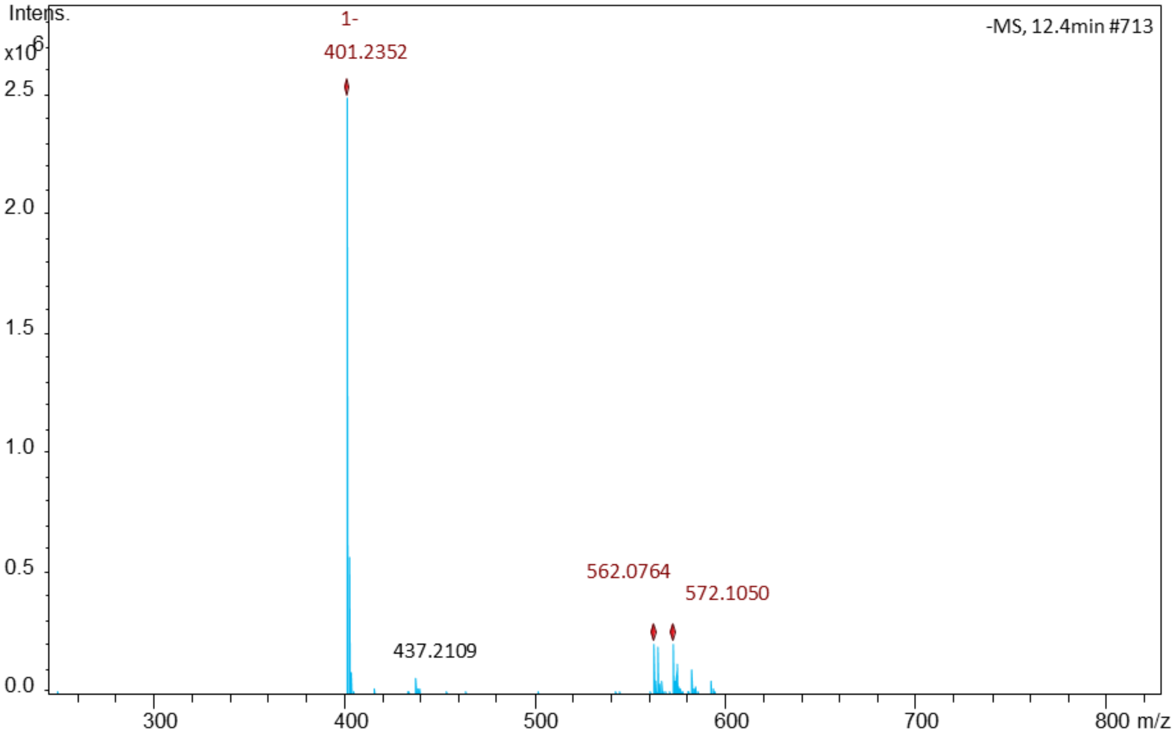

Fig. S25 HRESIMS spectrum of lucidone K (**3**)

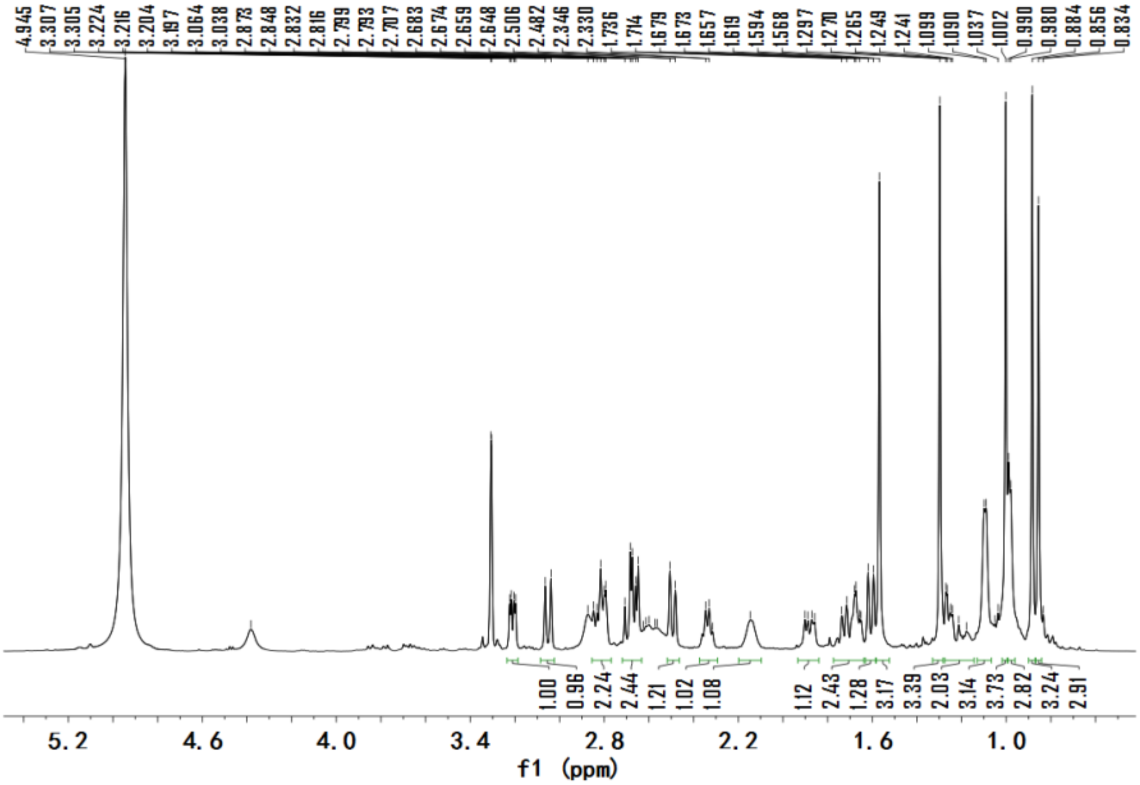

Fig. S26 ^{1}H NMR (600 MHz, CD_3OD) spectrum of lucidone L (**4**)

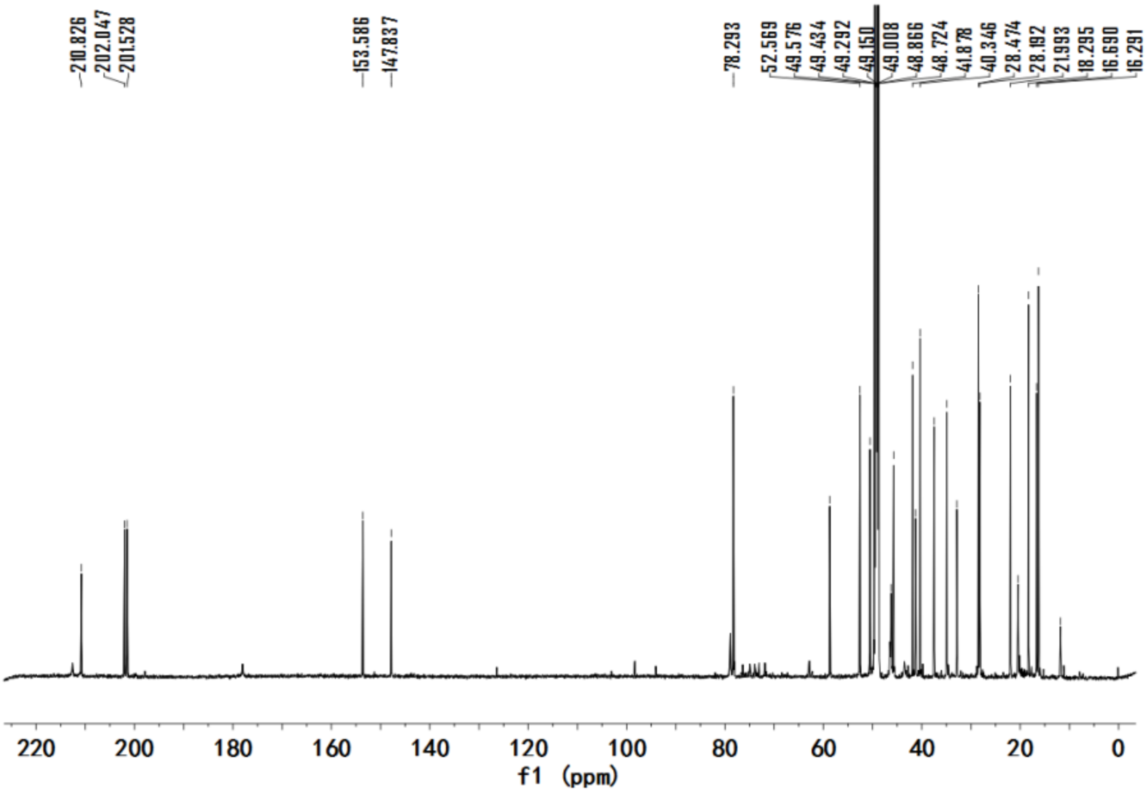

Fig. S27 ^{13}C NMR (150 MHz, CD_3OD) spectrum of lucidone L (**4**)

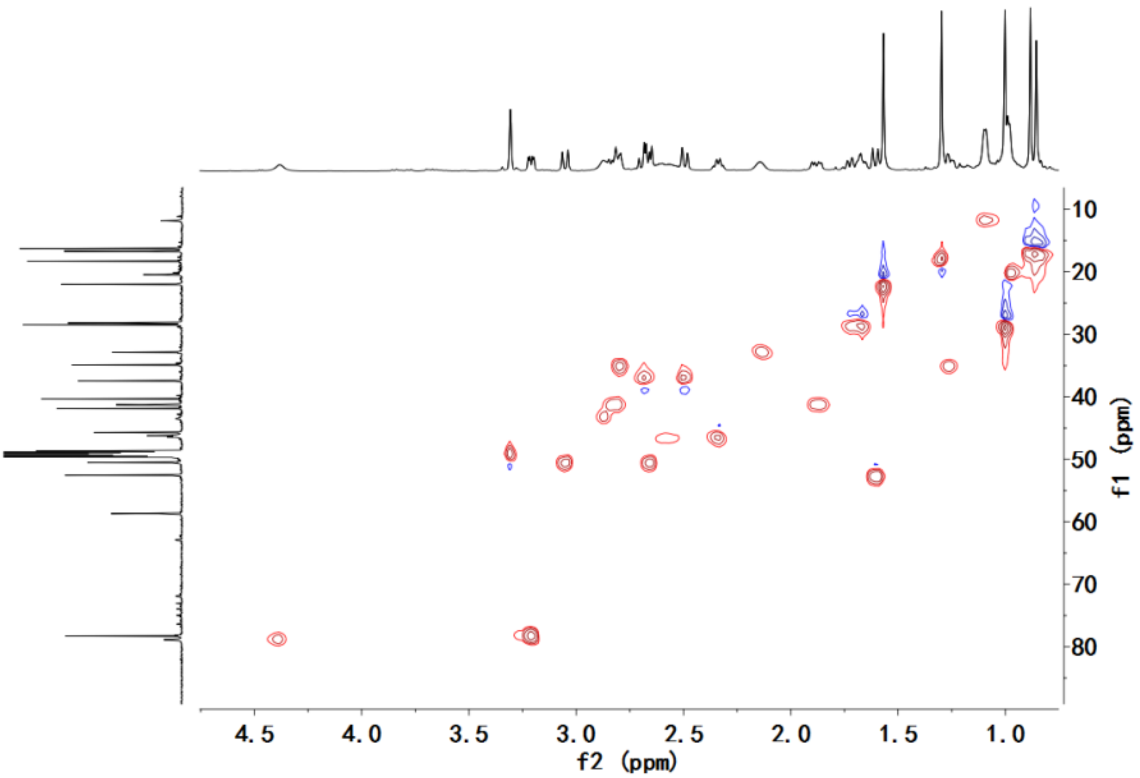

Fig. S28 HSQC (600 MHz, CD_3OD) spectrum of lucidone L (**4**)

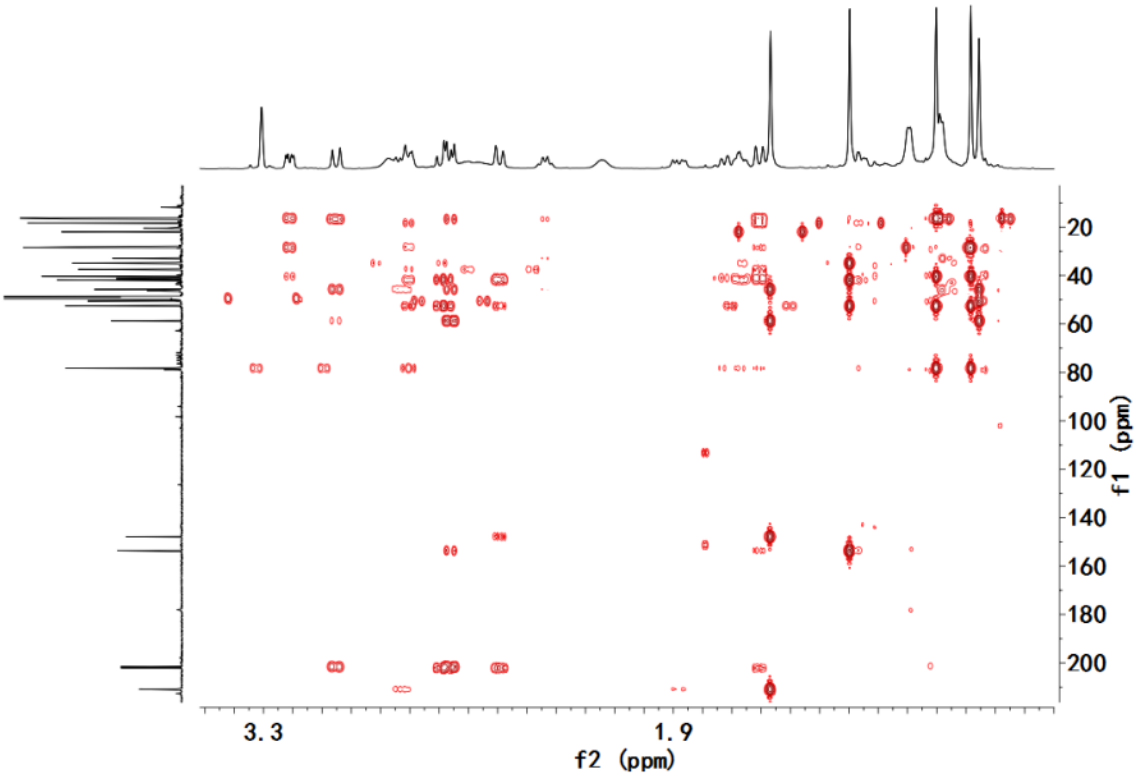

Fig. S29 HMBC (600 MHz, CD_3OD) spectrum of lucidone L (**4**)

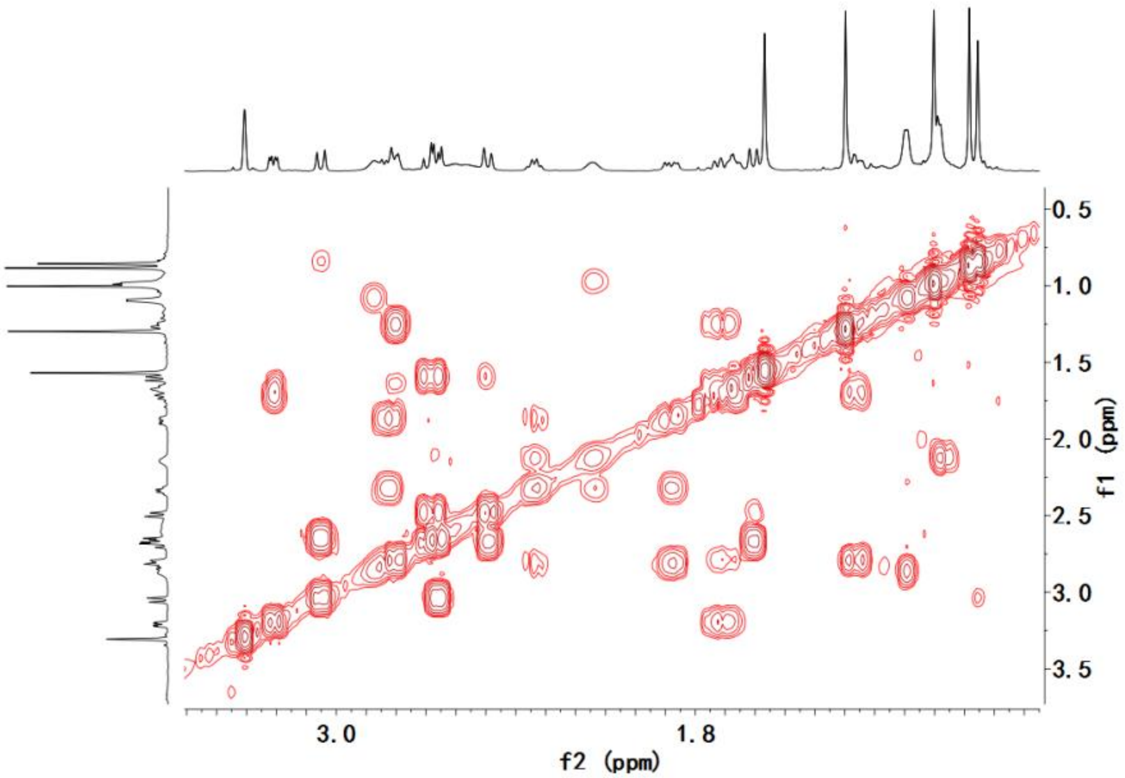

Fig. S30 ^{1}H-^{1}H COSY (600 MHz, CD_3OD) spectrum of lucidone L (**4**)

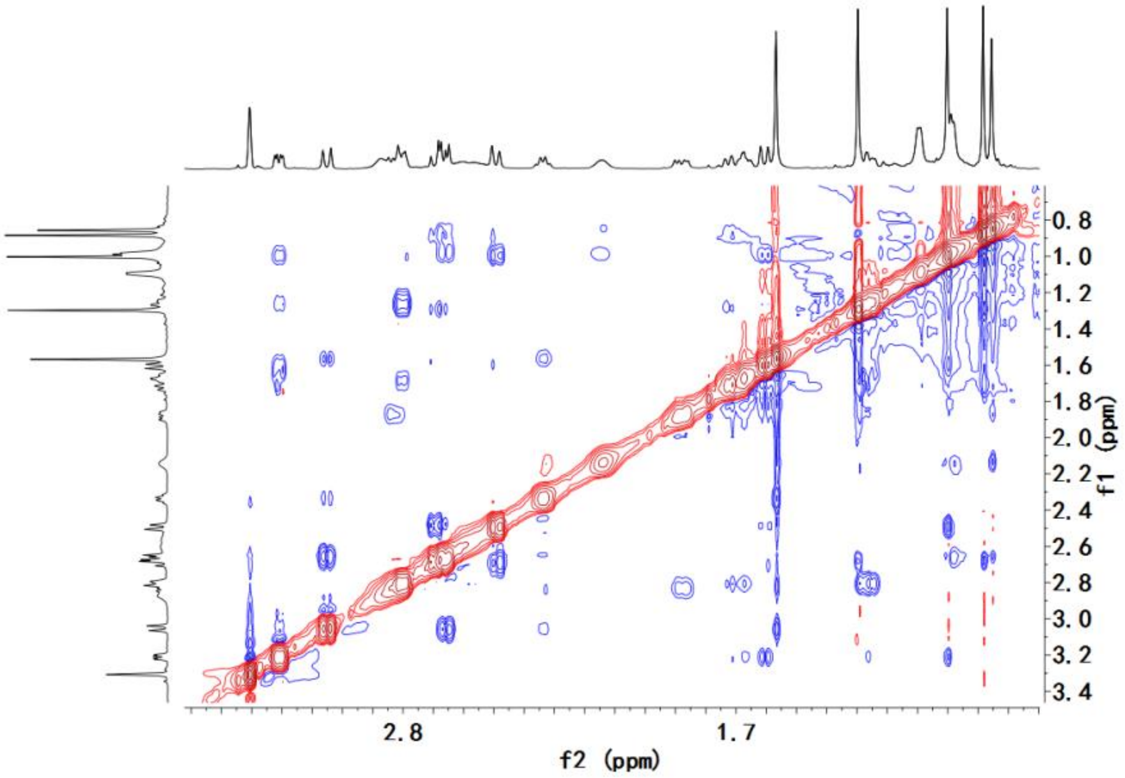

Fig. S31 ROESY (600 MHz, CD_3OD) spectrum of lucidone L (**4**)

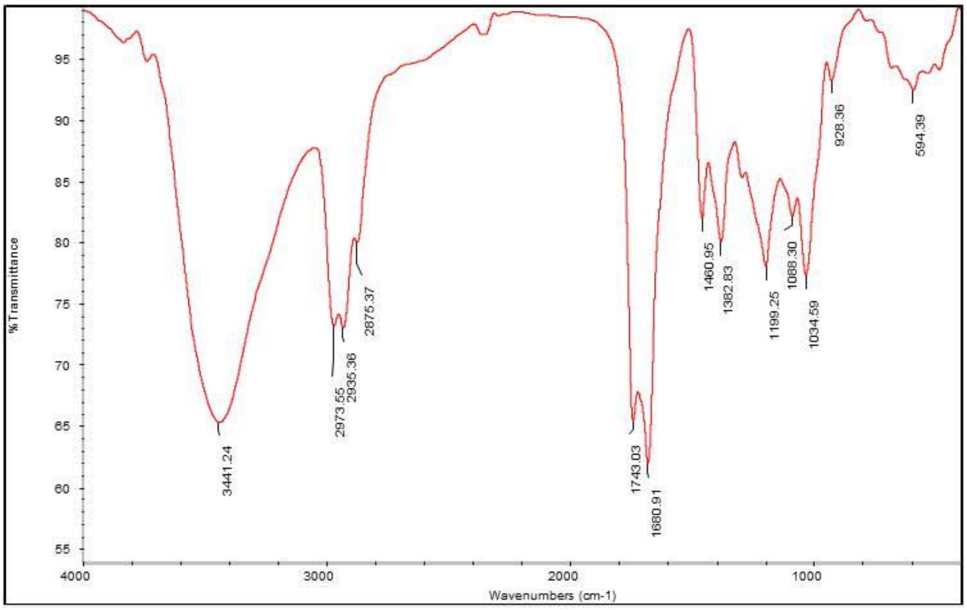

Fig. S32 IR spectrum of lucidone L (**4**)

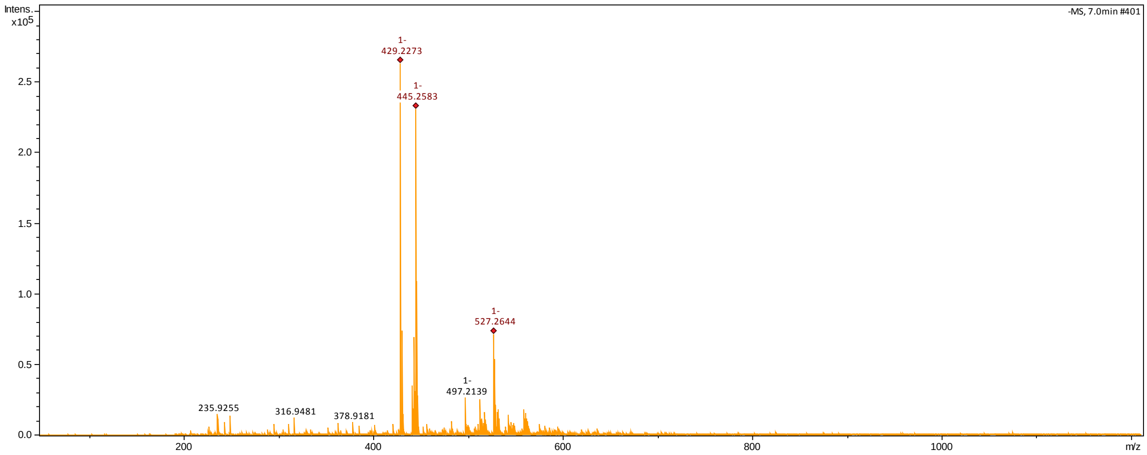

Fig. S33 HRESIMS spectrum of lucidone L (**4**)

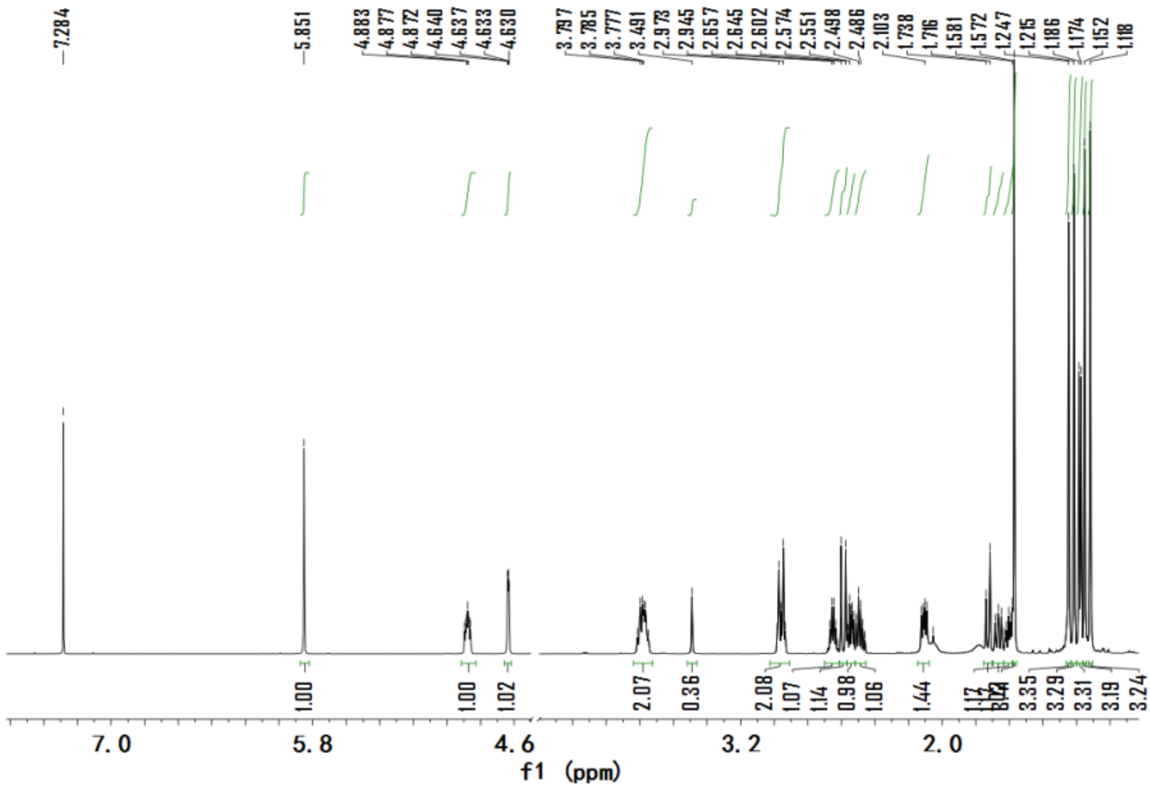

Fig. S34 ^{1}H NMR (600 MHz, $CDCl_3$) spectrum of ganosineniol B (**5**)

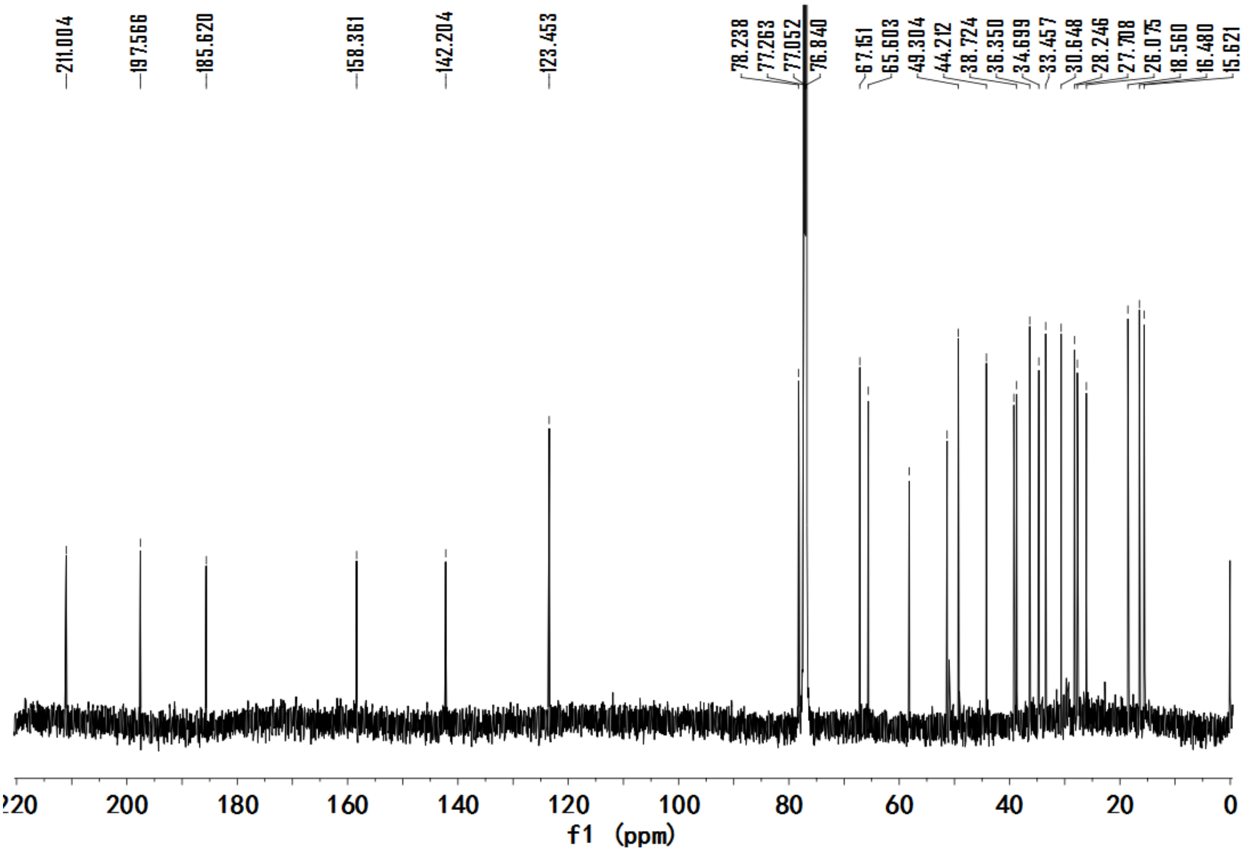

Fig. S35 ^{13}C NMR (150 MHz, $CDCl_3$) spectrum of ganosineniol B (**5**)

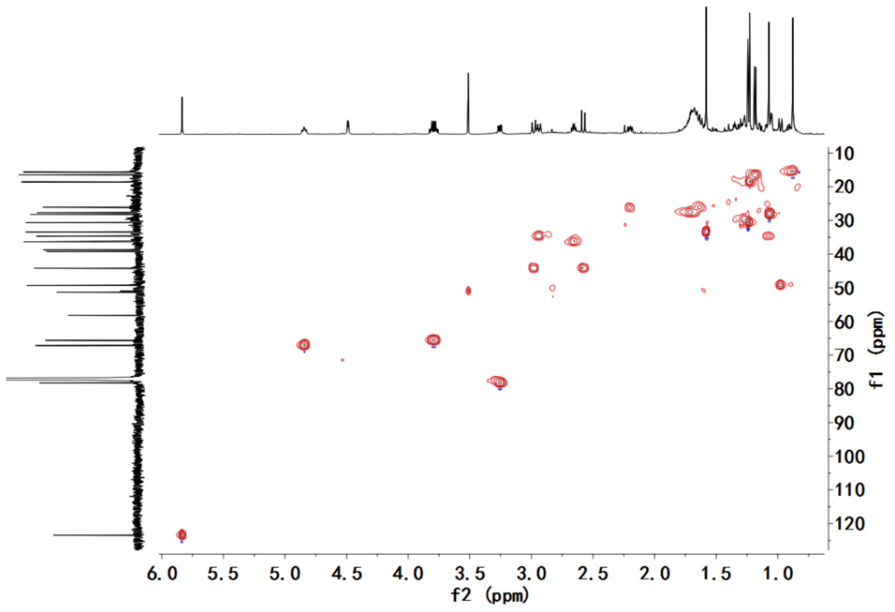

Fig. S36 HSQC (600 MHz, $CDCl_3$) spectrum of ganosineniol B (**5**)

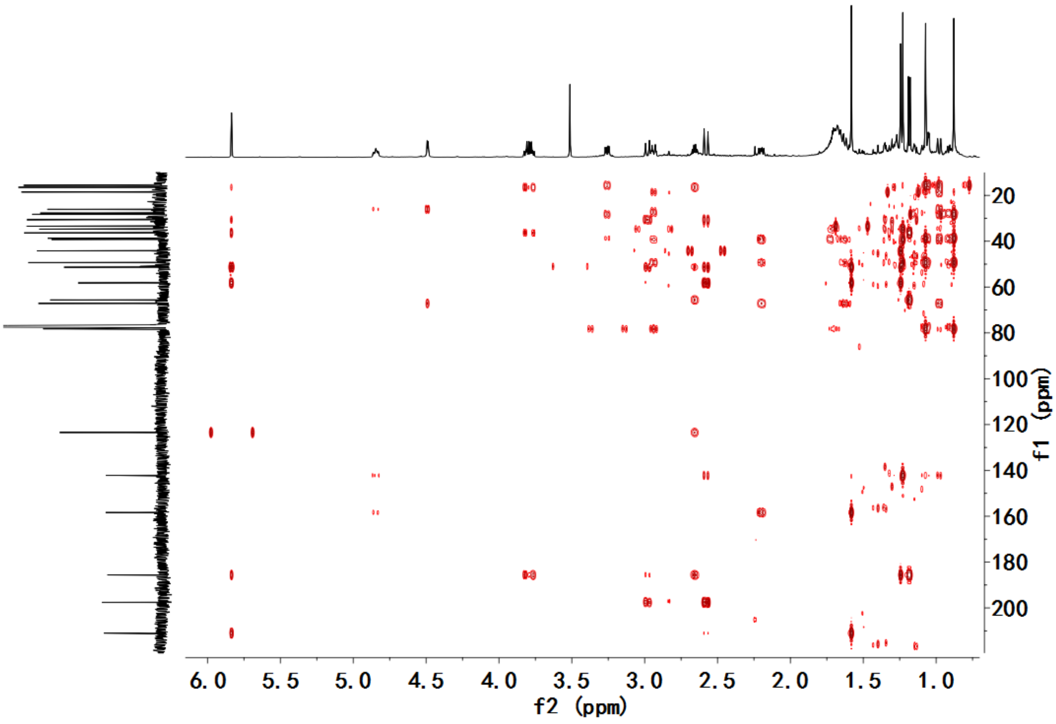

Fig. S37 HMBC (600 MHz, $CDCl_3$) spectrum of ganosineniol B (**5**)

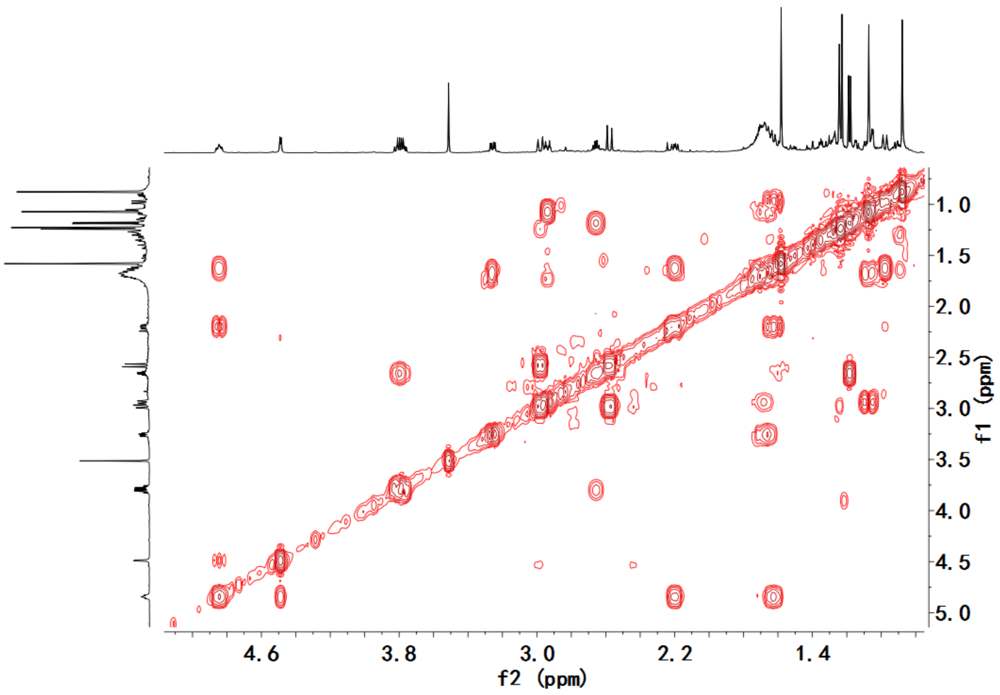

Fig. S38 ^{1}H-^{1}H COSY (600 MHz, $CDCl_3$) spectrum of ganosineniol B (**5**)

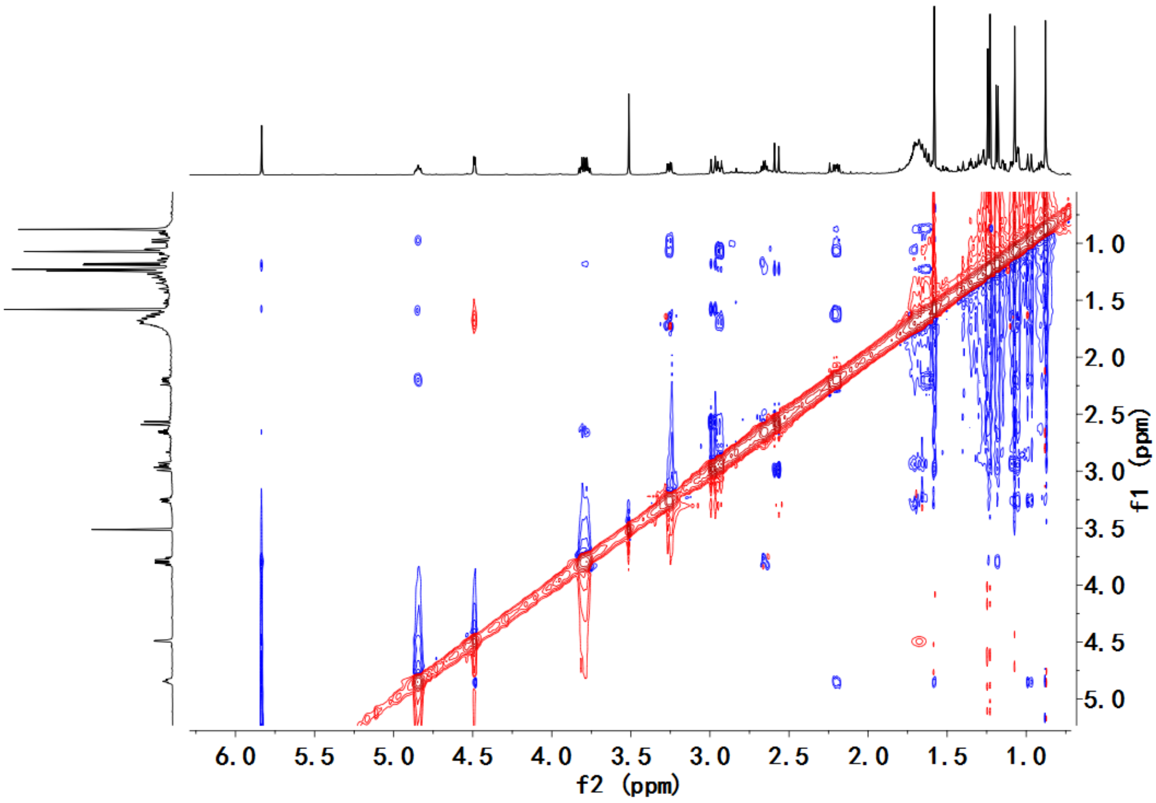

Fig. S39 ROESY (600 MHz, $CDCl_3$) spectrum of ganosineniol B (**5**)

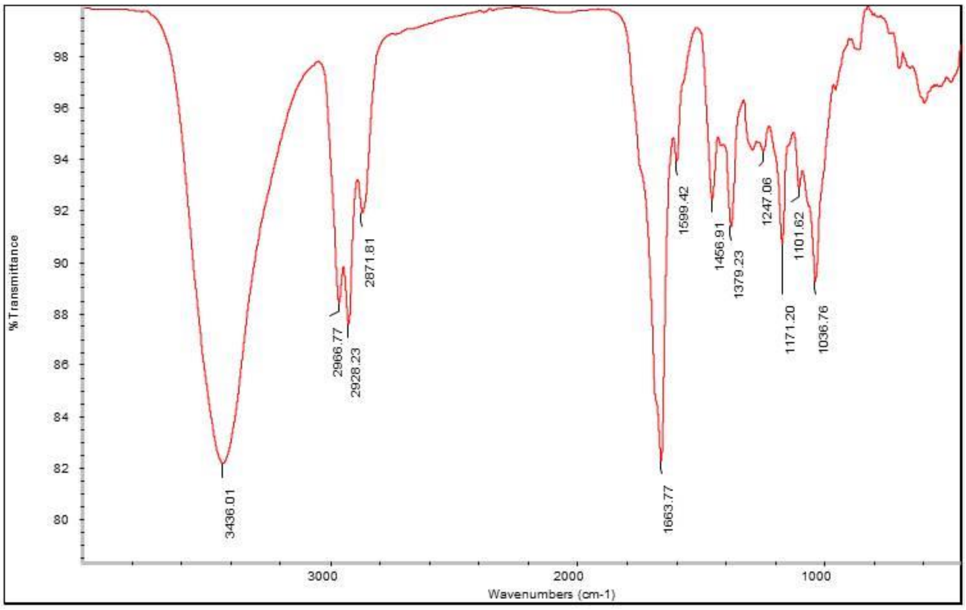

Fig. S40 IR spectrum of ganosineniol B (**5**)

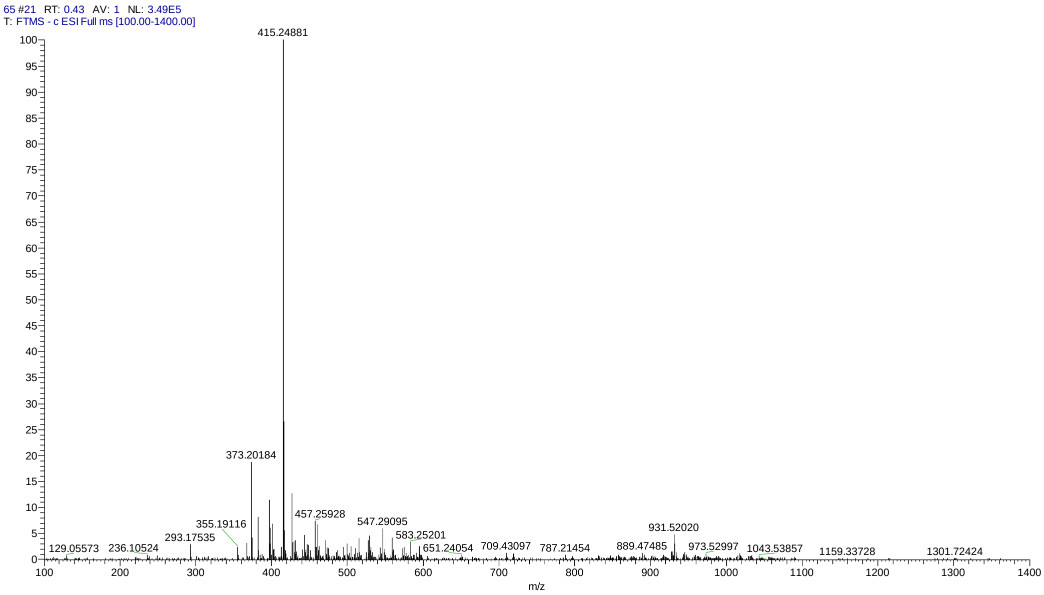

Fig. S41 HRESIMS spectrum of ganosineniol B (**5**)

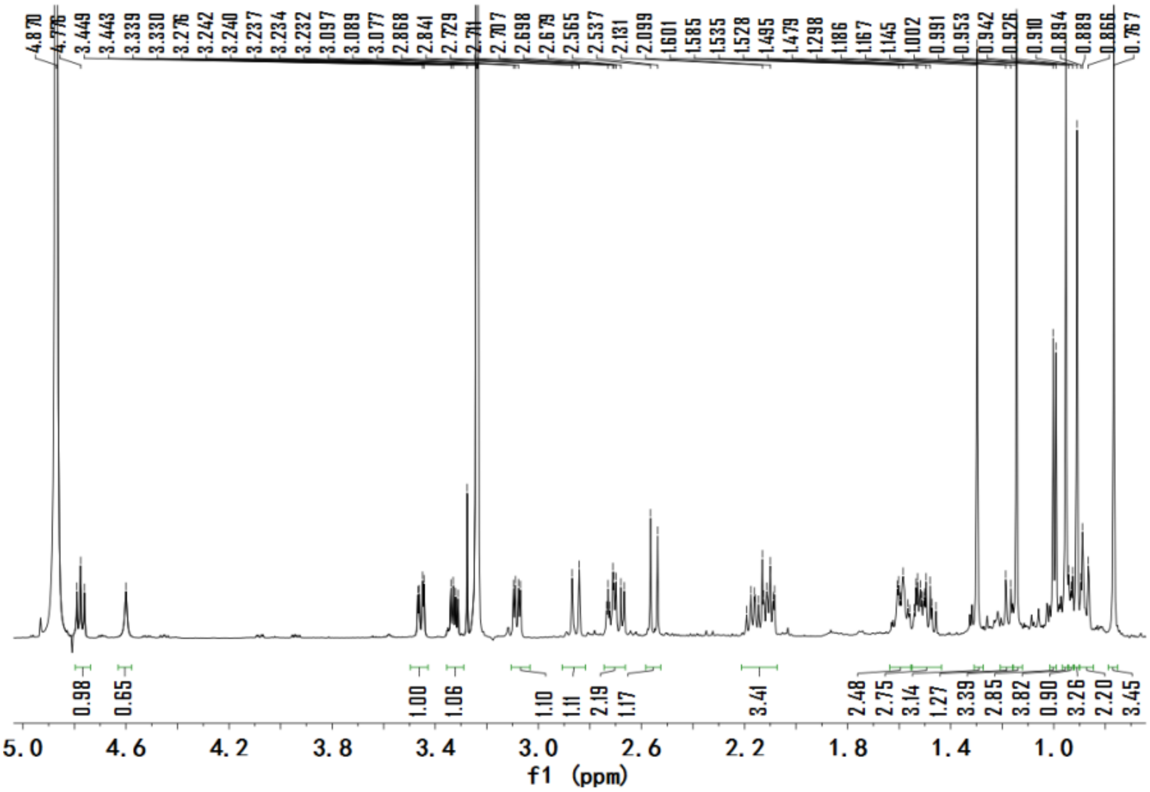

Fig. S42 ^{1}H NMR (600 MHz, CD_3OD) spectrum of ganosineniol C (**6**)

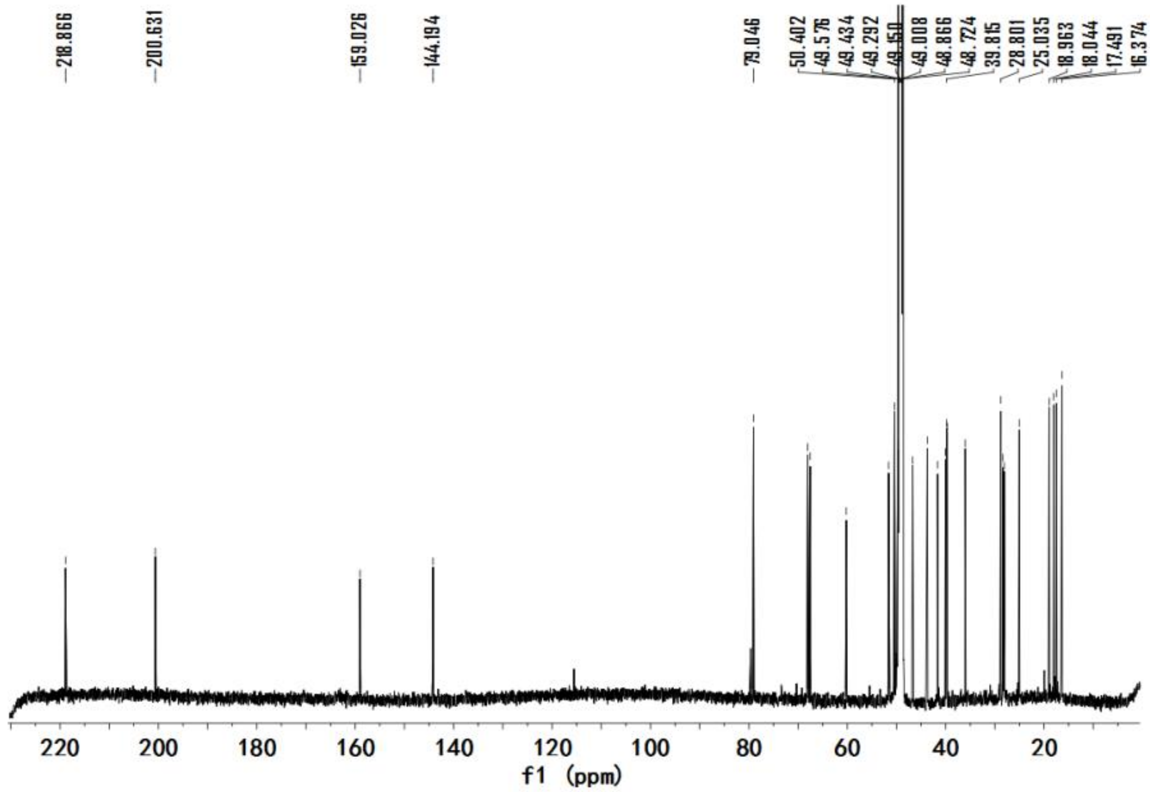

Fig. S43 ^{13}C NMR (150 MHz, CD_3OD) spectrum of ganosineniol C (**6**)

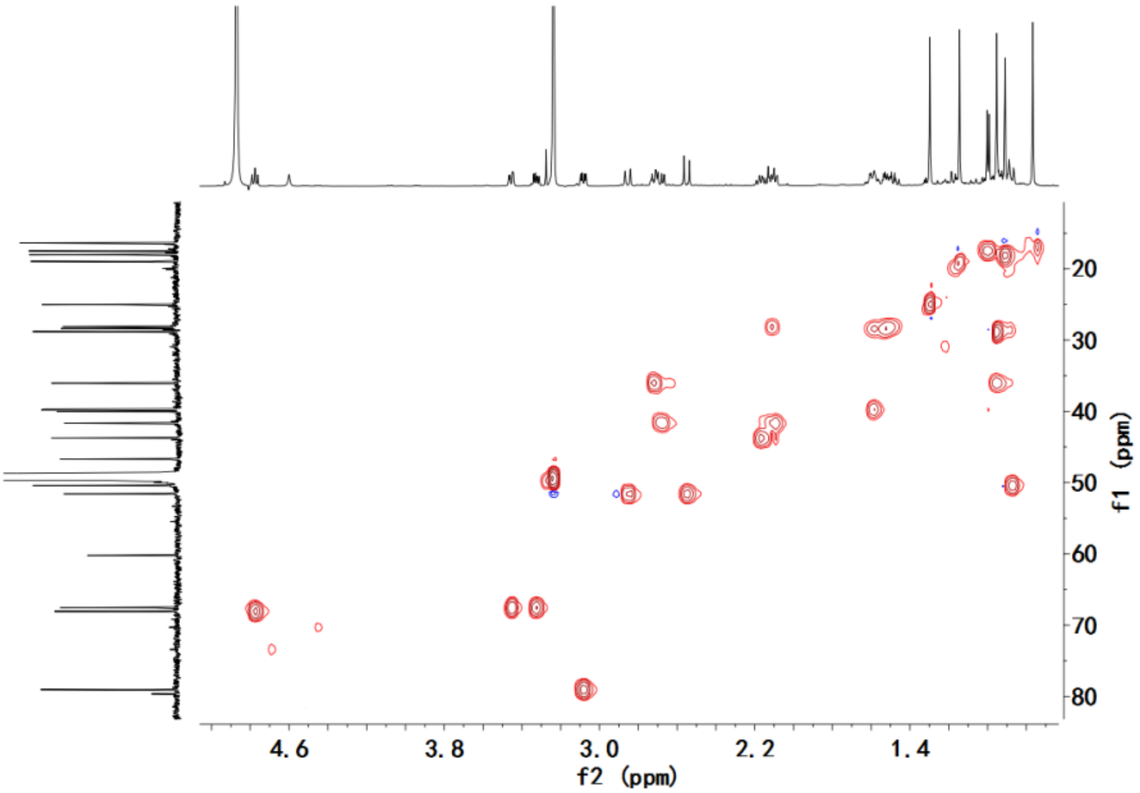

Fig. S44 HSQC (600 MHz, CD_3OD) spectrum of ganosineniol C (**6**)

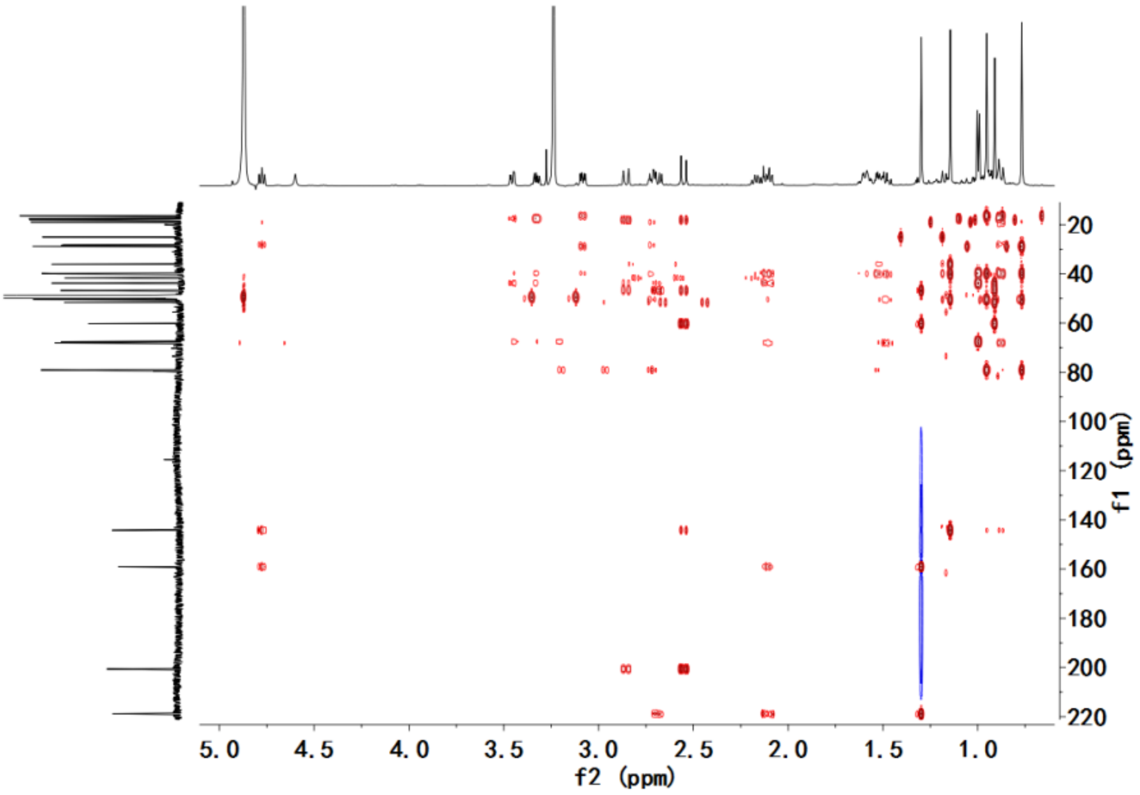

Fig. S45 HMBC (600 MHz, CD_3OD) spectrum of ganosineniol C (**6**)

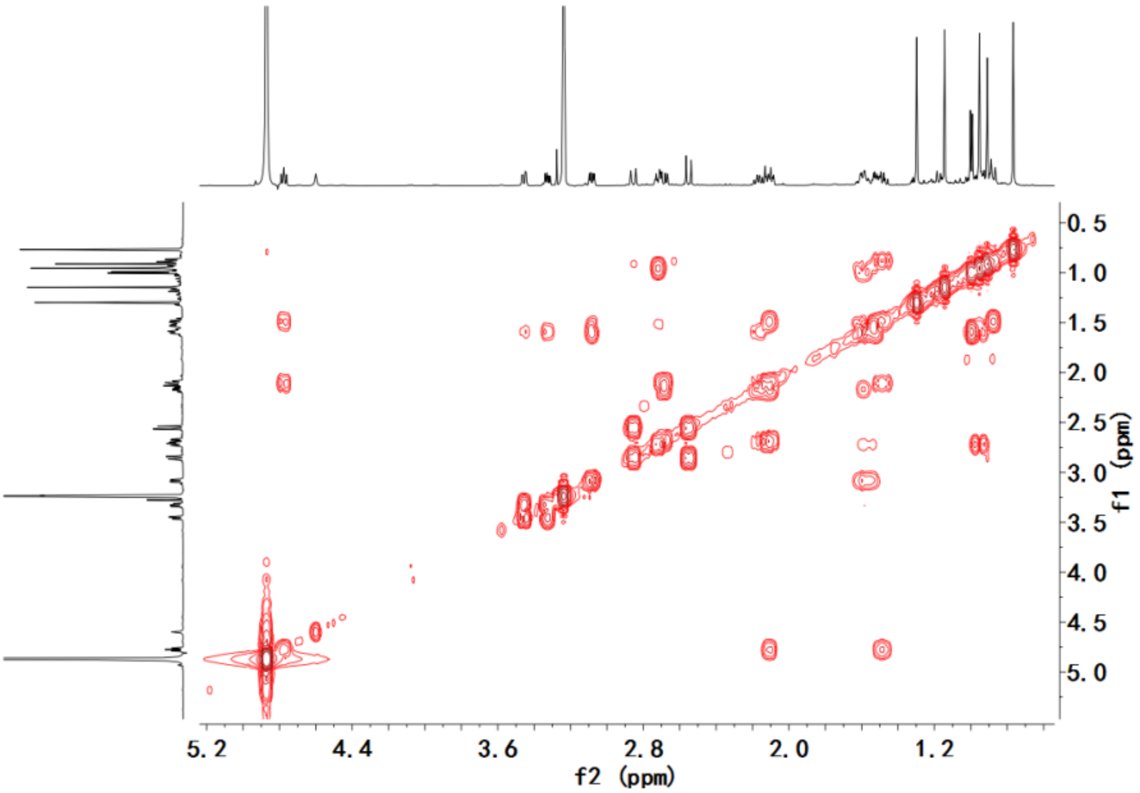

Fig. S46 ^{1}H-^{1}H COSY (600 MHz, CD_3OD) spectrum of ganosineniol C (**6**)

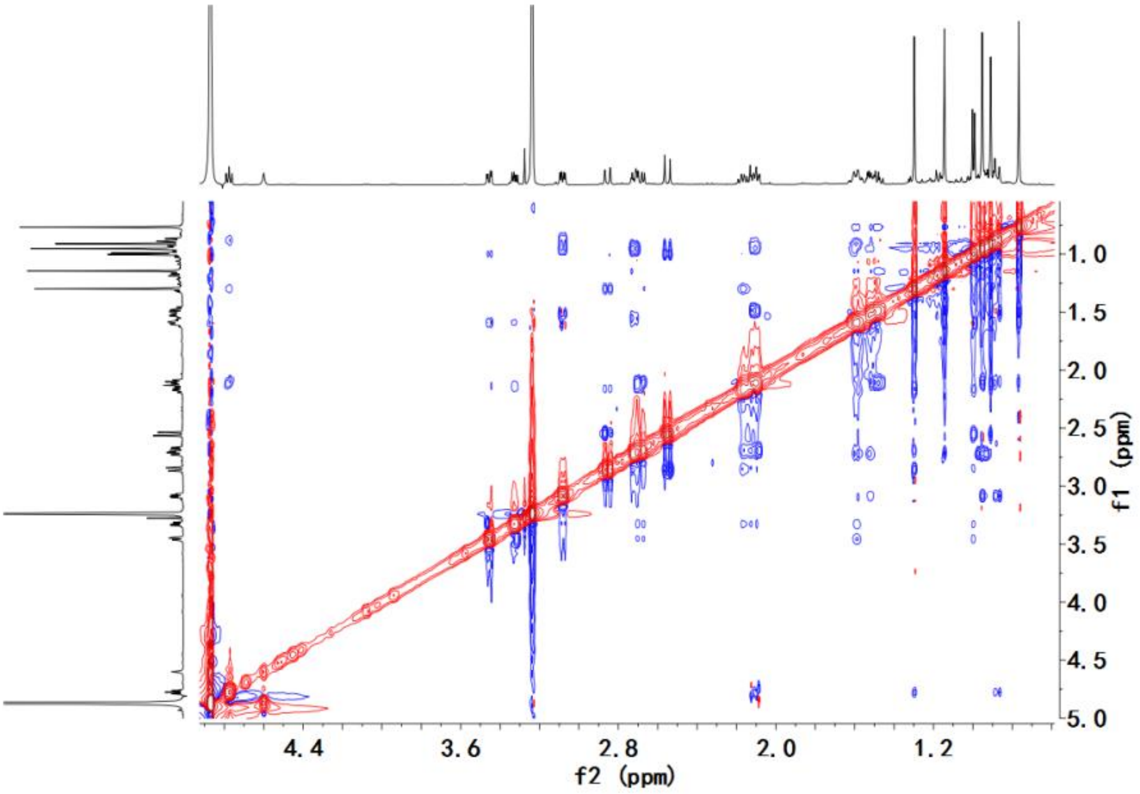

Fig. S47 ROESY (600 MHz, CD_3OD) spectrum of ganosineniol C (**6**)

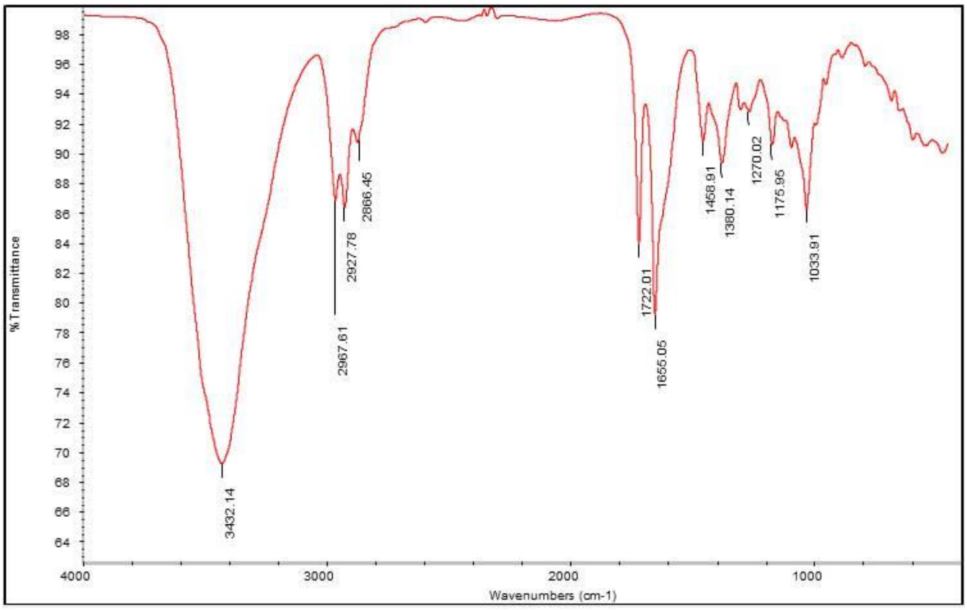

Fig. S48 IR spectrum of ganosineniol C (**6**)

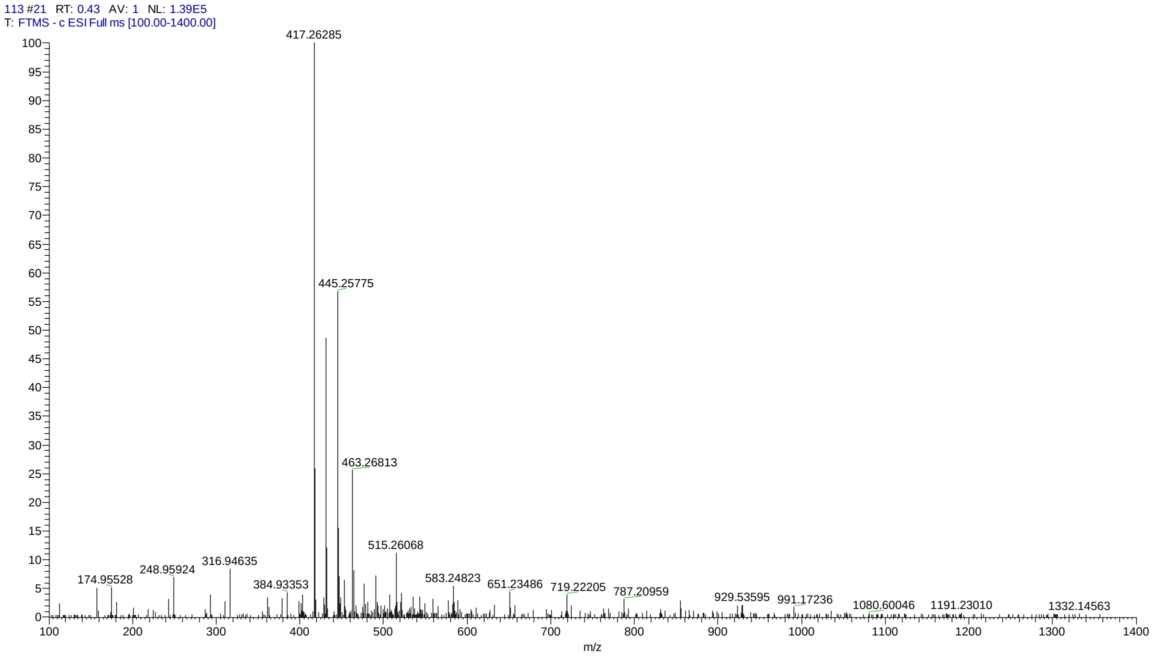

Fig. S49 HRESIMS spectrum of ganosineniol C (**6**)

Table S1 Inhibition rate of nortriterpenoids from *Ganoderma* at the concentration of 3 mM

**compounds**	**Inhibition rate (%)**
**3**	20.68
**4**	13.21
**7**	9.20
**8**	26.74
**9**	5.46

**10**	24.67
**11**	16.01
**12**	18.31
**13**	19.06
