# Supplementary figures and images for: Nortriterpenoids from the Fruiting Bodies of the Mushroom Ganoderma resinaceum
Source: Molecules. 2017 Jun 28;22(7):1073. doi: 10.3390/molecules22071073 (PMC6152414; doi:10.3390/molecules22071073)

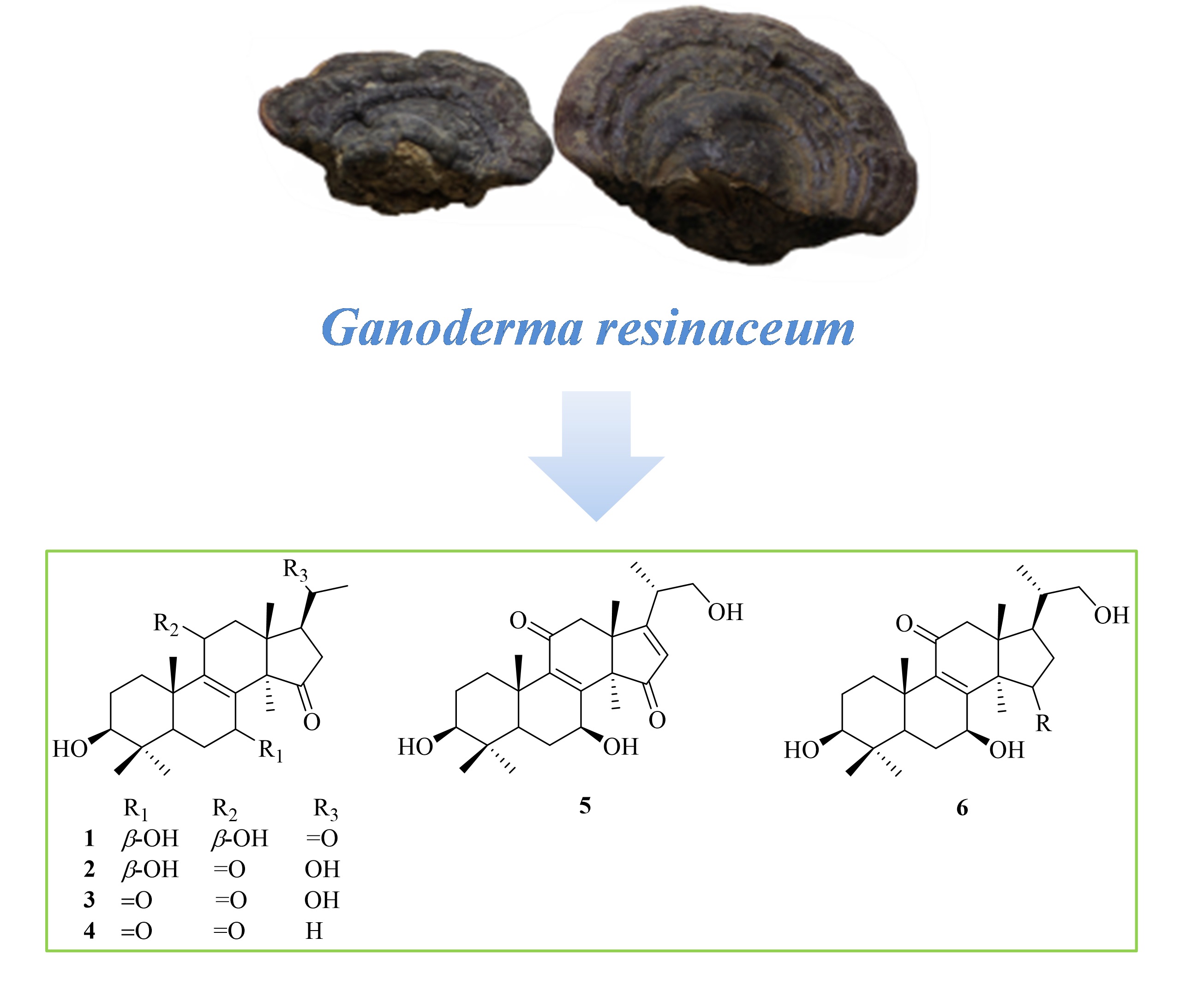

Supplement: Supplementary file 1 [file molecules-22-01073-s001.zip › molecules-203943-supplementary/molecules-203943-graphical.jpg]
